# TFPT Abundance Is Associated with Growth-Related Phenotypes and Cellular Features Linked to Lipid Peroxidation Defense in Hepatocellular Carcinoma

**DOI:** 10.3390/biology15161396

**Published:** 2026-08-14

**Authors:** Yueyue Guo, Yuting Zhou, Ziying Wu, Yilin Ma, Jingran Wang, Jizhe Zhou, Haifeng Li, Minlin Jiang, Delong Xie, Sangui Yi, Zongling Liu

**Affiliations:** 1 School of Basic Medical Sciences, Youjiang Medical University for Nationalities, Baise 533000, China; 2 Food Safety and Nutrition Experimental Teaching Demonstration Center, Department of Food Science and Engineering, Xinjiang Institute of Technology, Aksu 843000, China

**Keywords:** TFPT, hepatocellular carcinoma, lipid peroxidation defense, redox status, GPX4, FSP1

## Abstract

TCF3 fusion partner (TFPT) remains insufficiently characterized in hepatocellular carcinoma (HCC), particularly in relation to tumor growth and cellular features linked to lipid peroxidation defense. We integrated public transcriptomic and clinical datasets and established stable *TFPT*-knockdown and *TFPT*-overexpression models in MHCC97-H and Huh-7 cells. *TFPT* expression was higher in HCC tissues and selected HCC cell lines. In TCGA-LIHC, higher *TFPT* expression was associated with shorter overall survival after multivariable adjustment (HR, 1.37; 95% CI, 1.06–1.77), although this association was not reproduced in GSE14520. *TFPT*-high tumors were enriched for E2F Targets, MYC Targets, G2/M Checkpoint, and metabolic and cellular-stress-related programs. Internally constructed descriptive scores related to lipid peroxidation defense were higher in *TFPT*-high tumors but showed limited external reproducibility. In vitro, shTFPT cells showed lower CCK-8 signals, colony formation, monolayer closure, GSH levels, total glutathione peroxidase activity, and relative GPX4, SLC7A11, and FSP1 protein abundance, together with higher Fe^2+^ and MDA levels and higher BODIPY-C11 green-to-red fluorescence ratios; oeTFPT cells showed opposite patterns. These findings support an association between TFPT abundance and growth-related phenotypes and cellular features linked to lipid peroxidation defense in HCC, without establishing direct molecular regulation, ferroptotic cell death, causality, or independently validated prognostic utility.

## Simple Summary

Primary liver cancer remains difficult to treat, and tumor cells can grow rapidly while adapting to oxidative stress. We examined whether a poorly understood protein called TCF3 fusion partner (TFPT) is associated with these features. We analyzed public tumor datasets and experimentally reduced or increased TFPT levels in two liver cancer cell models. Tumors with higher *TFPT* expression showed stronger patterns related to cell division, metabolism, and cellular stress responses. In the cell models, reducing TFPT levels was associated with weaker growth-related activity and changes consistent with reduced protection against membrane lipid oxidation, whereas increasing TFPT levels produced the opposite pattern. However, the association between *TFPT* expression and patient survival observed in one dataset was not reproduced in a second dataset, and the experiments did not show that TFPT directly controls ferroptosis, an iron-dependent form of cell death. These findings suggest that TFPT is associated with a growth-related, stress-associated state in liver cancer and provide a basis for future studies of tumor growth and cellular responses to oxidative stress.

## 1. Introduction

Hepatocellular carcinoma (HCC) accounts for most cases of primary liver cancer and remains a major cause of cancer-related mortality worldwide [1,2]. Although targeted therapies and immune checkpoint inhibitors have expanded treatment options for advanced HCC, durable responses are achieved in only a subset of patients [2], underscoring the need to better understand the biological determinants of treatment response. Rapidly proliferating tumor cells also need to maintain redox homeostasis under increased metabolic and oxidative demands. Identifying factors associated with both proliferative activity and cellular redox status may therefore provide additional insight into HCC progression and treatment response.

Ferroptosis is an iron-dependent form of regulated cell death driven by excessive phospholipid peroxidation [3]. It occurs when lipid peroxide generation exceeds the capacity of cellular defense systems to detoxify these products. Iron metabolism, cysteine availability, glutathione metabolism, polyunsaturated fatty acid composition, and antioxidant capacity collectively influence ferroptosis susceptibility [3,4]. In HCC, these processes have been linked to tumor growth, metabolic regulation, and therapeutic response [4]. However, defense against lipid peroxidation is not determined by a single antioxidant component, because HCC cells can engage several partially overlapping defense systems.

The SLC7A11–GSH–GPX4 axis is a major glutathione-dependent system that limits phospholipid peroxidation. SLC7A11, the light-chain component of system xc^−^, mediates cystine uptake to support intracellular glutathione synthesis, whereas GPX4 uses reduced glutathione to detoxify phospholipid hydroperoxides [5,6]. Ferroptosis suppressor protein 1 (FSP1), encoded by *AIFM2*, provides a parallel defense mechanism by maintaining the reduced coenzyme Q_10_ pool independently of GPX4 [7,8]. Although these systems act through distinct biochemical mechanisms, they can collectively protect cells against lipid peroxidation. Consequently, changes in SLC7A11, GPX4, or FSP1 alone may not fully represent the broader cellular state associated with protection against lipid oxidation.

Multiple regulatory mechanisms have been reported to enhance cellular resistance to lipid peroxidation and ferroptotic stress in HCC, including transcriptional activation of *SLC7A11*, post-translational regulation of system xc^−^ activity, and stabilization of GPX4 [9,10,11]. These observations indicate that defense against lipid peroxidation is governed by interconnected regulatory networks rather than by a single linear pathway, which may contribute to context-dependent responses to pharmacological inhibition of system xc^−^. Imidazole ketone erastin (IKE) inhibits system xc^−^ and disrupts cystine-dependent glutathione synthesis, although cellular responses to IKE may vary across HCC contexts [5,12].

TCF3 fusion partner (*TFPT*), also known as FB1 or the TCF3/E2A fusion partner, has previously been implicated in cell-cycle and cell-death processes. *TFPT* was originally identified through its association with childhood precursor B-cell acute lymphoblastic leukemia [13]. Early ectopic-expression studies of the rat TFPT homolog Amida suggested possible roles in cell death and cell-cycle regulation [14,15]. Subsequent work on *TFPT* reported context-dependent effects on apoptosis that varied with cell type and culture density [16]. Most previous studies were performed in non-hepatic models, and the biological relevance of *TFPT* in solid tumors remains poorly defined. In particular, its expression pattern, association with patient survival, and relationship with cellular features linked to lipid peroxidation defense remain poorly characterized in HCC.

In this study, we investigated whether TFPT abundance is associated with growth-related phenotypes and cellular features linked to lipid peroxidation defense in HCC. Public transcriptomic and clinical datasets were analyzed to characterize *TFPT* expression, survival associations, and transcriptional programs related to proliferation, metabolism, and cellular stress. Stable *TFPT*-knockdown and *TFPT*-overexpression models in MHCC97-H and Huh-7 cells were used to assess growth-related phenotypes, biochemical indicators of redox status and lipid oxidation, and the relative abundance of selected proteins linked to lipid peroxidation defense. An exploratory IKE experiment using a single concentration and exposure duration was additionally performed in the *TFPT*-knockdown models to evaluate CCK-8 readouts under system xc^−^ inhibition. These analyses were intended to characterize *TFPT*-associated phenotypes rather than to establish direct molecular regulation, ferroptosis-specific sensitization, or ferroptotic cell death.

## 2. Materials and Methods

### 2.1. Public Datasets and Bioinformatics Analyses

To characterize *TFPT* expression and its clinical and transcriptional associations in hepatocellular carcinoma (HCC), publicly available transcriptomic profiles were integrated with the corresponding clinical records. RNA-sequencing data for The Cancer Genome Atlas Liver Hepatocellular Carcinoma cohort (TCGA-LIHC) were retrieved from the National Cancer Institute Genomic Data Commons (NCI GDC) [17,18]. Gene-level STAR-TPM output generated by the GDC STAR Counts workflow using GENCODE v36 annotation was used. When multiple annotated features corresponded to the same gene symbol, their TPM values were summed before transformation. Gene expression was expressed as log_2_(sum TPM + 1).

The primary expression comparison included 371 HCC tissues and 50 adjacent non-tumor liver tissues. Fifty matched tumor–non-tumor pairs were additionally evaluated using a paired design. *TFPT* expression was independently examined in the normalized GSE14520-GPL3921 cohort obtained from the Gene Expression Omnibus (GEO) [19,20]. This external evaluation cohort included 225 HCC tissues and 220 non-tumor liver tissues, and probe set 218996_at was used to represent *TFPT*.

Clinical outcome information for TCGA-LIHC was obtained from the Legacy clinical matrix hosted by UCSC Xena [21]. The median *TFPT* expression level among tumor samples was used to assign patients to *TFPT*-high and *TFPT*-low groups. Kaplan–Meier analysis and univariable Cox proportional-hazards regression were performed in 365 patients with available overall survival information, including 130 death events. Multivariable analyses were restricted to 339 patients with complete covariate information and 114 death events and were adjusted for age, sex, American Joint Committee on Cancer stage, and histologic grade. *TFPT* was entered into the Cox models either as a median-defined binary variable or as a continuous variable. In the continuous model, the hazard ratio represented a one-unit increase in log_2_(sum TPM + 1). Records lacking any required covariate were excluded using a complete-case approach.

The survival association of *TFPT* was additionally evaluated in GSE14520 using samples profiled on the GPL3921 and GPL571 platforms. *TFPT* expression was standardized within each platform before Cox modeling. The primary platform-adjusted model included 225 complete cases with 86 death events and was adjusted for age, sex, tumor–node–metastasis stage, and microarray platform. The hazard ratio represented a one-standard-deviation increase in within-platform standardized *TFPT* expression. Seventeen additional patients had available *TFPT* expression and survival information but lacked usable stage information and were therefore excluded from the primary multivariable model. As a sensitivity analysis, all 242 patients with available *TFPT* expression and survival information, including 96 death events, were analyzed using a platform-stratified Cox model with standardized *TFPT* expression as the sole covariate. Microarray platform was used as the stratification factor, allowing separate baseline hazards for the GPL3921 and GPL571 platforms. The proportional-hazards assumption was evaluated using rank-transformed scaled Schoenfeld residuals for the individual covariates and the global model. These analyses were regarded as external exploratory evaluations rather than as confirmatory prognostic validation.

For transcriptome-wide differential-expression analysis, the 371 TCGA-LIHC tumors were divided into *TFPT*-low (*n* = 186) and *TFPT*-high (*n* = 185) groups according to the median *TFPT* expression level. Welch’s *t* statistic was calculated independently for each gene, followed by Benjamini–Hochberg correction for multiple testing. Genes were considered differentially expressed when the false discovery rate (FDR) *q* value was < 0.05 and the absolute difference in mean log_2_-transformed expression between the two groups was ≥0.5.

Preranked gene set enrichment analysis was performed using the prerank function in GSEApy v1.2.1 [22,23]. Genes were ranked according to the corresponding Welch’s *t* statistics comparing *TFPT*-high and *TFPT*-low tumors. Ties were resolved according to gene symbol without the addition of random jitter. The analysis used 1000 permutations and a fixed random seed of 20260712. The complete Hallmark gene set collection from Molecular Signatures Database release 2026.1.Hs was evaluated [24]. Enrichment was summarized using the normalized enrichment score, nominal *p* value, and FDR *q* value. Selected proliferation-, metabolism-, and cellular-stress-related gene sets are displayed in the main figure. All significantly enriched Hallmark gene sets are shown in Supplementary Figure S4, whereas the complete output for all 50 Hallmark gene sets is provided in Supplementary Table S7.

A descriptive proliferation index was calculated using the following 19 genes: *MKI67*, *PCNA*, *TOP2A*, *MCM2*, *MCM3*, *MCM5*, *MCM6*, *CDK1*, *CCNB2*, *CDC20*, *BIRC5*, *AURKA*, *AURKB*, *PLK1*, *UBE2C*, *E2F8*, *MYBL2*, *RRM2*, and *MAD2L1*. Within the 371 TCGA-LIHC tumors, the expression value of each gene was independently standardized to a *z* score. The sample-level proliferation index was then calculated as the unweighted mean of the standardized expression values. Because this gene panel was assembled and evaluated within the same *TFPT*-focused transcriptomic dataset, it was interpreted as a descriptive expression summary rather than as independent validation of the differential-expression or enrichment results.

Four additional expression-based composite scores were generated using the same gene-wise standardization and equal-weight averaging strategy. These comprised a ferroptosis-suppressor-related score, a GPX4-GSH-defense-related score, an FSP1-CoQ_10_-defense-related score, and an overall lipid-peroxidation-defense-related score. All constituent genes contributed in the same direction, no reverse scoring was applied, and *TFPT* was excluded from all four scores. Supplementary Table S2 provides the constituent genes, assigned biological functions, scoring directions, supporting references, and overlap between the proliferation and lipid-peroxidation-defense-related panels.

Score composition was informed by foundational ferroptosis studies and FerrDb V2 [5,6,7,8,25]. Previously reported HCC prognostic signatures based on ferroptosis-related genes were reviewed for biological context but were not used to define the present scores [26,27]. Spearman rank-correlation analysis was additionally performed to examine associations between *TFPT* expression and 37 prespecified ferroptosis-related genes. The composite scores were intended to summarize relative expression patterns and were not interpreted as direct measurements of ferroptosis, pathway activity, cellular function, or molecular regulation by *TFPT*.

To assess whether differences in the composite scores were disproportionately driven by individual constituent genes, leave-one-gene-out sensitivity analyses were performed. Each score was recalculated repeatedly after removing one constituent gene at a time. *TFPT*-high and *TFPT*-low tumors were compared using two-sided Wilcoxon rank-sum tests, and effect sizes were summarized using Cliff’s delta. The direction and magnitude of each leave-one-gene-out result were compared with those obtained using the corresponding complete score. Benjamini–Hochberg correction was applied separately within each score and, in a complementary global analysis, across all leave-one-gene-out tests. These results are presented in Supplementary Figure S5.

The reproducibility of the four expression-based composite scores was additionally evaluated in GSE14520 using tumor samples profiled on the GPL3921 and GPL571 platforms. Microarray probes were mapped to gene symbols. When multiple probes represented the same gene, the probe with the highest median absolute deviation among tumor samples was selected. This selection criterion was independent of the association between the probe and *TFPT*. Gene expression was standardized within each platform, and each score was calculated as the unweighted mean of the available constituent genes. The number and proportion of represented genes were recorded for each score and platform. After score calculation, *TFPT* expression and each resulting score were standardized within each platform before regression modeling.

Associations between standardized *TFPT* expression and standardized score values were evaluated in 247 GSE14520 tumors with available expression data using platform-adjusted linear models. The standardized score was specified as the outcome, whereas standardized *TFPT* expression and platform were included as predictors. *TFPT*-by-platform interaction terms were examined in separate models to evaluate potential platform-dependent associations. Benjamini–Hochberg correction was applied across the four primary *TFPT*–score associations and separately across the four platform-interaction tests. These external analyses were considered evaluations of reproducibility rather than formal validation of the custom scores. Gene-coverage information and model results are presented in Supplementary Figure S6.

Score compositions, sample-level scores, complete correlation results, full Hallmark enrichment output, sensitivity-analysis results, and external-evaluation source data are provided in the Supplementary Materials and associated bioinformatics source-data files.

As an additional method-harmonization sensitivity analysis, the TCGA-LIHC scores were recalculated using only the score genes represented on each GSE14520 platform, and platform-specific FSP1–CoQ_10_-defense-related associations and leave-one-gene-out analyses were examined to assess the influence of gene coverage and individual score components (Supplementary Figure S6).

### 2.2. Cell Culture and Generation of Stable Cell Models

MHCC97-H (Cat. No. C5108), Huh-7 (Cat. No. C5560), HepG2 (Cat. No. C5175), and THLE-2 (Cat. No. C5664) cells were obtained from Zhejiang Baidi Biotechnology Co., Ltd. (Hangzhou, China). The immortalized hepatic epithelial cell line THLE-2 was included as a nonmalignant reference. MHCC97-H, Huh-7, and HepG2 cells were cultured in high-glucose Dulbecco’s modified Eagle medium (Wuhan Servicebio Technology Co., Ltd., Wuhan, China; Cat. No. G4511) supplemented with 10% fetal bovine serum (Beyotime Biotechnology, Shanghai, China; Cat. No. C0252) and 1% penicillin–streptomycin (Beyotime, Cat. No. C0222). THLE-2 cells were maintained in the manufacturer-provided complete THLE-2 culture medium (Zhejiang Baidi Biotechnology Co., Ltd., Cat. No. C5664-500). All cells were cultured at 37 °C in a humidified atmosphere containing 5% CO_2_.

Stable polyclonal MHCC97-H and Huh-7 cell models with reduced or increased *TFPT* expression were generated using a piggyBac transposon system adapted from a previously reported procedure [28]. The complete *TFPT* coding sequence was inserted into PB-flag to generate the overexpression construct, whereas one *TFPT*-targeting short hairpin RNA sequence was inserted into PB-U6P to generate the knockdown construct. Cells were seeded into six-well plates at 5 × 10^5^ cells per well. After attachment, each well was transfected with 500 ng of the corresponding piggyBac plasmid and 500 ng of the Super PiggyBac transposase plasmid using Lipo8000 transfection reagent (Beyotime, Cat. No. C0533). Empty PB-flag and PB-U6P carrying a non-targeting short hairpin RNA were used as the overexpression and knockdown controls, respectively.

Puromycin selection was initiated 48 h after transfection at a concentration of 2.0 μg/mL and continued for 7 days. The surviving polyclonal cell populations were retained for subsequent experiments. Changes in *TFPT* transcript and protein levels were verified by RT-qPCR and Western blotting, respectively. The oligonucleotide sequences used for construct preparation, quantitative PCR, and *TFPT* silencing are provided in Supplementary Table S1.

### 2.3. RNA Extraction and RT-qPCR

Total cellular RNA was extracted using the HiPure Total RNA Mini Kit (Magen Biotechnology Co., Ltd., Guangzhou, China; Cat. No. R4111-02) according to the manufacturer’s instructions. RNA concentration and purity were measured using a TGem Plus spectrophotometer (TIANGEN Biotech (Beijing) Co., Ltd., Beijing, China). First-strand complementary DNA was synthesized using BeyoRT III Premix with gDNA EZeraser (Beyotime, Cat. No. D7185S).

Quantitative PCR was performed using BeyoFast SYBR Green qPCR Mix (Beyotime, Cat. No. D7260), with *GAPDH* used as the internal reference. The thermal cycling protocol consisted of an initial denaturation step at 95 °C for 30 s, followed by 40 cycles of denaturation at 95 °C for 15 s and annealing/extension at 60 °C for 30 s. Amplicon specificity was evaluated by post-amplification melting-curve analysis.

Relative transcript expression was calculated using the 2^−ΔΔCt^ method. For each sample, ΔCt was calculated as Ct_TFPT − Ct_GAPDH. For comparisons among THLE-2 and the HCC cell lines, THLE-2 served as the calibrator. For the *TFPT*-knockdown models, the corresponding sh-Ctrl group within each cell line served as the calibrator, whereas for the *TFPT*-overexpression models, the corresponding Vector control group within each cell line served as the calibrator. ΔΔCt was calculated as ΔCt_sample − ΔCt_calibrator, and relative expression was expressed as 2^−ΔΔCt^. Within each independent biological experiment, technical-replicate values were averaged before statistical analysis. ΔCt values were used for inferential statistical testing, whereas 2^−ΔΔCt^ values were used for graphical presentation. Primer sequences are provided in Supplementary Table S1.

### 2.4. Western Blotting and Densitometric Analysis

Total cellular protein was extracted on ice using RIPA lysis buffer (Beyotime, Cat. No. P0013B) supplemented with 1 mM phenylmethylsulfonyl fluoride (Beyotime, Cat. No. ST506). Cell lysates were centrifuged at 12,000× *g* for 15 min at 4 °C, and the resulting supernatants were collected. Protein concentrations were determined using a BCA Protein Assay Kit (Beyotime, Cat. No. P0009).

For each lane, 25 μg of total protein was separated using 12% sodium dodecyl sulfate–polyacrylamide gel electrophoresis and transferred onto 0.45-μm polyvinylidene fluoride membranes (Servicebio, Cat. No. G6050-0.45) by wet electroblotting at 400 mA for 25 min. Before transfer, the membranes were activated in methanol. Nonspecific binding was blocked for 25 min at room temperature using 1× QuickBlock Protein-Free Blocking Buffer (Beyotime, Cat. No. P0242). The membranes were subsequently incubated with the corresponding primary antibodies overnight at 4 °C.

After primary-antibody incubation, the membranes were washed three times with Tris-buffered saline containing Tween 20 for 5 min per wash. Depending on the host species of the primary antibody, the membranes were incubated for 1 h at room temperature with horseradish-peroxidase-conjugated goat anti-rabbit IgG (Servicebio, Cat. No. GB23303; 1:15,000) or horseradish-peroxidase-conjugated goat anti-mouse IgG (Servicebio, Cat. No. GB23301; 1:15,000). The membranes were then washed three additional times with Tris-buffered saline containing Tween 20 for 5 min per wash.

High-sensitivity enhanced chemiluminescence substrate (Servicebio, Cat. No. G2074) was used for loading-control detection, whereas target-protein bands were developed using ultrasensitive enhanced chemiluminescence reagent (Servicebio, Cat. No. G2020). Chemiluminescent signals were acquired using a Tanon imaging system (Tanon Science & Technology Co., Ltd., Shanghai, China). For each target protein, groups included in the same direct comparison were developed using the same chemiluminescent substrate and acquired under unchanged imaging settings.

The primary antibodies used were directed against TFPT (1:2000; Taizhou Baijia Biotechnology Co., Ltd., Taizhou, China; Cat. No. IPB13735), GPX4 (1:7500; Proteintech Group, Inc., Rosemont, IL, USA; Cat. No. 82822-2-RR), SLC7A11 (1:4500; Proteintech, Cat. No. 82115-2-RR), FSP1 (1:4000; Proteintech, Cat. No. 20886-1-AP), β-actin (1:50,000; Proteintech, Cat. No. 66009-1-Ig), and GAPDH (1:250,000; Proteintech, Cat. No. 60004-1-Ig). β-actin or GAPDH was used as the loading control, as indicated in the corresponding figures.

Western blot densitometry was performed using ImageJ v1.54. Three independent biological experiments were quantified. For each sample, the background-subtracted intensity of the target-protein band was divided by the intensity of the corresponding β-actin or GAPDH band. The resulting target/loading-control ratios from all biological replicates were normalized to the mean value of the corresponding control group, which was set to 1.0.

For comparisons of TFPT protein abundance among THLE-2 and the three HCC cell lines, normalized values were analyzed using one-way analysis of variance followed by Dunnett’s multiple-comparisons test, with THLE-2 used as the reference group. For the stable cell models, two-group comparisons were performed separately within each cell line and experimental model using two-sided paired Student’s *t* tests, with pairing by independent biological experiment. Knockdown and overexpression samples were obtained from different membranes and were therefore quantified and statistically analyzed as separate experiments. No direct densitometric comparison was made between the knockdown and overexpression models or between groups processed on different membranes.

Representative immunoblots are presented in the main figures. Densitometric results from three independent biological experiments, including individual data points, mean ± standard deviation, and statistical comparisons, are presented in Supplementary Figures S7–S9. The corresponding full-length, uncropped Western blot images, including molecular-weight information and lane identities, are provided separately in Supplementary File S2.

### 2.5. CCK-8, Colony-Formation, and Scratch-Wound Assays

Cell-associated metabolic activity was assessed using the Cell Counting Kit-8 assay. MHCC97-H and Huh-7 cells were seeded into 96-well plates at 2000 cells per well, with independent wells prepared for measurements at 0, 24, 48, and 72 h. The baseline measurement was performed 4 h after seeding. At each designated time point, 10 μL of CCK-8 reagent (UElandy Inc., Suzhou, China; Cat. No. C6005M) was added to each well. After incubation for 2 h at 37 °C, absorbance was measured at 450 nm.

For colony-formation assays, MHCC97-H and Huh-7 cells were seeded into six-well plates at 700 cells per well and cultured for 14 days. The culture medium was replaced every 3 days. Colonies were fixed with 600 μL of 4% paraformaldehyde (Servicebio, Cat. No. G1101) for 30 min and stained with 600 μL of 0.1% crystal violet (Beyotime, Cat. No. C0121) for 30 min at room temperature. The plates were then washed, air-dried, and photographed. Colonies containing at least 50 cells were counted using ImageJ under identical analysis criteria for all groups.

For scratch-wound assays, 1 × 10^6^ cells were seeded into each well of a six-well plate and cultured overnight until a confluent monolayer was formed. A straight wound was generated using a sterile 10-μL pipette tip. Detached cells were removed by washing with phosphate-buffered saline, after which serum-free medium was added. Images were acquired immediately after scratching and at 24 and 48 h.

Residual wound areas were measured using ImageJ v1.54. Monolayer closure was expressed as the percentage reduction in wound area relative to the initial wound area at 0 h. Because wound-area reduction may result from both cell proliferation and cell displacement, this assay was interpreted as a measure of monolayer closure rather than as a direct measurement of cell migration.

### 2.6. Measurement of Biochemical Readouts Associated with Redox Status and Lipid Oxidation

Biochemical readouts associated with cellular redox status and lipid oxidation were evaluated by measuring malondialdehyde (MDA), reduced glutathione (GSH), total glutathione peroxidase (GPx) activity, and ferrous iron (Fe^2+^) using commercial colorimetric assay kits from Servicebio according to the manufacturer’s instructions. Total protein concentration was measured using the BCA method, and all biochemical measurements were normalized to total protein content. MDA, GSH, and Fe^2+^ levels were expressed as nmol/mg protein, whereas total GPx activity was expressed as U/mg protein. The GPx assay measured total glutathione peroxidase activity and was not specific to GPX4.

For MDA measurement, approximately 1 × 10^7^ cells were lysed on ice in 200 μL of IP lysis buffer (Servicebio, Cat. No. G2038). The lysates were centrifuged at 10,000× *g* for 10 min at 4 °C. The resulting supernatants were reacted with the thiobarbituric acid substrate provided in the MDA Assay Kit (Servicebio, Cat. No. G4300). Absorbance was measured at 532 nm, and MDA levels were calculated using the corresponding standard curve.

For GSH, total GPx activity, and Fe^2+^ measurements, 1 × 10^6^ cells were used for each assay. Cells were homogenized in the corresponding extraction medium, consisting of phosphate-buffered saline for the GSH and total GPx assays and the kit-supplied extraction buffer for the Fe^2+^ assay. Samples were centrifuged at 10,000× *g* for 10 min at 4 °C. The resulting supernatants were incubated with the corresponding chromogenic reagents from the GSH Assay Kit (Servicebio, Cat. No. G4305), total GPx Assay Kit (Servicebio, Cat. No. G4310; DTNB-based), or Fe^2+^ Assay Kit (Servicebio, Cat. No. G4323).

Absorbance was measured at 412 nm for the GSH and total GPx assays and at 593 nm for the Fe^2+^ assay. For Fe^2+^ measurement, the reaction mixture was incubated in the dark for 40 min at 37 °C.

### 2.7. BODIPY-C11 Staining and IKE-Associated Stress Assay

Cellular lipid oxidation was evaluated using the BODIPY-C11 581/591 fluorescent probe (Beyotime, Cat. No. S0043S). Cells were seeded into 96-well plates at 5 × 10^4^ cells per well. After attachment, the cells were incubated with 5 μM BODIPY-C11 for 20 min at 37 °C in the dark. The staining solution was subsequently removed, and the cells were gently washed twice with phosphate-buffered saline to reduce background fluorescence. Fresh phosphate-buffered saline was added before fluorescence acquisition.

Within each independent experiment, representative fluorescence images for all groups were acquired using identical acquisition settings. The oxidized and nonoxidized forms of BODIPY-C11 were visualized as green and red fluorescence, respectively. Green and red fluorescence intensities were measured using a Spark multimode microplate reader (Tecan Austria GmbH, Grödig, Austria). The BODIPY-C11 green-to-red fluorescence ratio was calculated as (green fluorescence intensity/red fluorescence intensity) × 100.

The association between *TFPT* depletion and the CCK-8 response under imidazole ketone erastin (IKE) exposure was examined in stable sh-Ctrl and shTFPT cells. IKE was purchased from Shanghai Macklin Biochemical Co., Ltd. (Shanghai, China; Cat. No. I795246; 1 mL) and prepared as a 10 mM stock solution in dimethyl sulfoxide (DMSO). Cells were seeded into 96-well plates at 5000 cells per well and allowed to attach overnight. Based on preliminary experiments showing a measurable response in HCC cells following IKE treatment, 40 μM IKE and a 24-h exposure duration were selected for the subsequent exploratory experiment. Treatment with 40 μM IKE resulted in a final DMSO concentration of 0.4% (*v*/*v*). Vehicle-control wells received the same final concentration of DMSO without IKE.

Following treatment, 10 μL of CCK-8 reagent was added to each well. After incubation for 2 h at 37 °C, absorbance at 450 nm was measured. The assay was repeated in five independent biological experiments, with five technical wells included for each condition within each experiment. The five technical-well values were averaged before statistical analysis, and the mean value from each independent biological experiment constituted the statistical unit. Individual technical wells were not treated as independent observations.

Raw biological-replicate mean OD450 values from the four experimental conditions—sh-Ctrl plus DMSO, sh-Ctrl plus IKE, shTFPT plus DMSO, and shTFPT plus IKE—were used for the primary interaction analysis. Each independent biological experiment simultaneously included all four conditions. Raw biological-replicate mean OD450 values were analyzed separately for each cell line using two-way repeated-measures analysis of variance, with independent biological experiment used as the matching factor across the four experimental conditions. The *TFPT*-status-by-treatment interaction was used as the primary test of whether the effect of IKE differed according to *TFPT* status.

As a secondary exploratory analysis, an IKE/DMSO-normalized value was calculated separately for the sh-Ctrl and shTFPT conditions within each independent biological experiment by dividing the mean OD450 value of the IKE-treated condition by that of the corresponding DMSO-treated condition. The normalized values were compared between sh-Ctrl and shTFPT cells using a two-sided unpaired Student’s *t* test.

This exploratory experiment evaluated a single IKE concentration and one exposure duration and was restricted to the *TFPT*-knockdown models. It was designed to evaluate CCK-8 readouts under system xc^−^ inhibition rather than to establish ferroptotic cell death or ferroptosis-specific sensitization.

### 2.8. Statistical Analysis

Bioinformatics analyses were performed using Python v3.12.13 and R v4.5.3. The lifelines package v0.30.3 was used for Cox proportional-hazards regression and proportional-hazards diagnostics. GSEApy v1.2.1 was used for preranked enrichment analysis, and pandas v2.3.3 and NumPy v2.4.6 were used for data handling. Kaplan–Meier plots were generated using the survival package v3.8-6 in R. Cell-based data and Western blot densitometry were analyzed using GraphPad Prism v10.1.2.

Unless otherwise stated, cell-based assays and Western blot densitometry were performed in three independent biological experiments. An independent biological experiment was defined as a separately initiated cell-culture experiment in which cells were independently seeded, cultured, and processed on a different occasion. Two technical replicates per condition were included within each biological experiment for RT-qPCR, time-course CCK-8, scratch-wound, biochemical, and BODIPY-C11 measurements. The IKE-associated assay was performed in five independent biological experiments, with five technical wells included for each condition within each experiment. Technical-replicate values were averaged within each experiment before statistical analysis, and the independent biological experiment constituted the statistical unit. Colony-formation assays were also conducted in three independent biological experiments. Data are presented as individual biological-replicate values with mean ± standard deviation. All *p* values were two-sided where applicable.

For transcriptomic comparisons not otherwise specified, the Wilcoxon rank-sum test was used for independent two-group comparisons, whereas matched tumor and adjacent non-tumor tissues were compared using the Wilcoxon signed-rank test. Transcriptome-wide differential-expression testing was performed using Welch’s *t* statistics as described in Section 2.1. Differences among histologic grades were evaluated using the Kruskal–Wallis test. Overall survival curves were estimated using the Kaplan–Meier method and compared using the log-rank test. Cox proportional-hazards regression was used to estimate hazard ratios and 95% confidence intervals. Proportional-hazards assumptions were evaluated using rank-transformed scaled Schoenfeld residuals for the individual covariates and global model tests.

The proliferation index was compared between *TFPT*-high and *TFPT*-low tumors using a two-sided Wilcoxon rank-sum test. The same test was applied separately to the four lipid-peroxidation-defense-related expression scores, followed by Benjamini–Hochberg correction across the four comparisons. Effect sizes for the complete expression scores were summarized using Cliff’s delta with 95% confidence intervals. Associations between *TFPT* expression and the 37 prespecified ferroptosis-related genes were evaluated using Spearman correlation coefficients, with Benjamini–Hochberg correction applied across the 37 tests.

For the leave-one-gene-out sensitivity analyses, each recalculated score was compared between *TFPT*-high and *TFPT*-low tumors using a two-sided Wilcoxon rank-sum test. Effect sizes were reported using Cliff’s delta. Benjamini–Hochberg correction was applied separately within each score and, in a complementary global analysis, across all leave-one-gene-out tests.

For the external GSE14520 score evaluation, standardized score values were modeled as continuous outcomes using linear regression, with standardized *TFPT* expression and platform specified as predictors. Separate models additionally included a *TFPT*-by-platform interaction term. Benjamini–Hochberg correction was applied across the four primary *TFPT*–score associations and separately across the four platform-interaction tests.

For planned two-group comparisons in cell-based experiments, analyses were performed separately within each cell line and experimental model using two-sided unpaired Student’s *t* tests. This approach was applied to the planned two-group comparisons for RT-qPCR, colony formation, monolayer closure, biochemical measurements, and BODIPY-C11 readouts. *TFPT* transcript expression in THLE-2 and the three HCC cell lines was compared using one-way analysis of variance followed by Dunnett’s multiple-comparisons test, with THLE-2 used as the reference group.

Time-course CCK-8 data were analyzed separately for the knockdown and overexpression experiments within each cell line using ordinary two-way analysis of variance, with time and *TFPT* status specified as factors. Planned shTFPT-versus-sh-Ctrl and oeTFPT-versus-Vector comparisons at individual time points were adjusted using the Šídák multiple-comparisons procedure.

For Western blot densitometry, the target-protein/loading-control ratio was normalized to the corresponding control-group mean before statistical analysis. Knockdown and overexpression experiments were analyzed separately because they were performed using different membranes. Two-group Western blot comparisons within each cell line and experimental model were performed using two-sided paired Student’s *t* tests, with pairing by independent biological experiment. Comparisons of TFPT protein abundance among THLE-2 and the three HCC cell lines were performed using one-way analysis of variance followed by Dunnett’s multiple-comparisons test.

For the IKE-associated assay, the five technical-well measurements for each condition were averaged within each of five independent biological experiments. Each independent experiment simultaneously included sh-Ctrl plus DMSO, sh-Ctrl plus IKE, shTFPT plus DMSO, and shTFPT plus IKE conditions. Raw biological-replicate mean OD450 values were analyzed separately for each cell line using two-way repeated-measures ANOVA, with independent biological experiment used as the matching factor across the four experimental conditions. The *TFPT*-status-by-treatment interaction was used as the primary test of whether the effect of IKE differed according to *TFPT* status. As a secondary exploratory analysis, IKE/DMSO-normalized values were compared between sh-Ctrl and shTFPT cells using a two-sided unpaired Student’s *t* test.

Statistical significance was defined as *p* < 0.05 or FDR *q* < 0.05 when correction for multiple testing was applied.

## 3. Results

### 3.1. TFPT Expression Was Higher in HCC Tissues and Selected HCC Cell Lines

**Figure 1:**
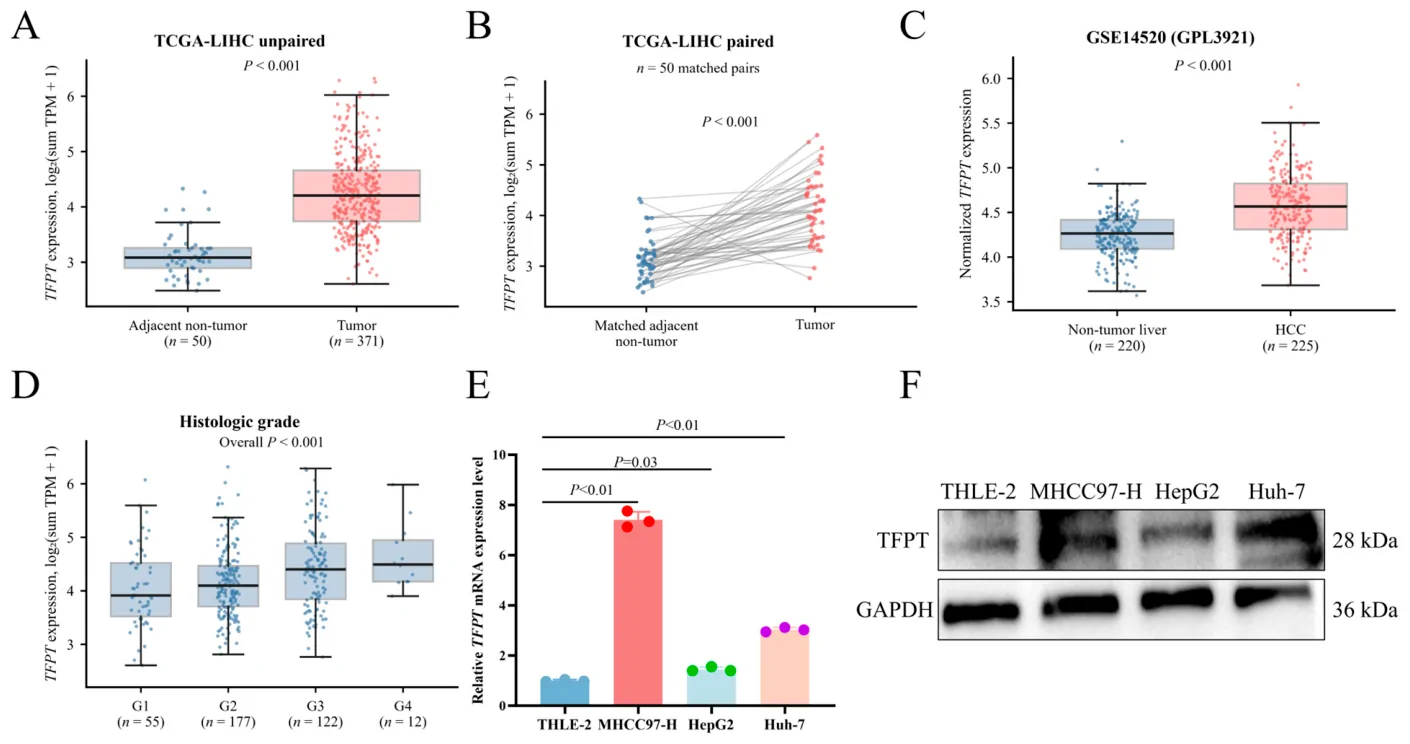
*TFPT* expression in HCC tissues and selected HCC cell lines. (**A**) *TFPT* transcript abundance in adjacent non-tumor liver tissues (*n* = 50) and primary HCC tissues (*n* = 371) from TCGA-LIHC. (**B**) Paired comparison of *TFPT* expression in 50 matched HCC and adjacent non-tumor liver samples. (**C**) Independent evaluation of *TFPT* expression in non-tumor liver tissues (*n* = 220) and HCC tissues (*n* = 225) from the GSE14520-GPL3921 cohort. (**D**) *TFPT* expression across TCGA-LIHC histologic grades: G1 (*n* = 55), G2 (*n* = 177), G3 (*n* = 122), and G4 (*n* = 12). Five tumors with missing grade information were excluded. (**E**) Relative *TFPT* mRNA expression measured by RT-qPCR in THLE-2, MHCC97-H, HepG2, and Huh-7 cells. (**F**) Representative TFPT immunoblot in THLE-2, MHCC97-H, HepG2, and Huh-7 cells; GAPDH served as the loading control. Densitometric analysis from three independent biological experiments is presented in Supplementary Figure S7, and the corresponding full-length, uncropped images are provided in Supplementary File S2. Panels (**A**,**C**,**D**) show individual observations overlaid on boxplots, whereas paired samples in panel (**B**) are connected by lines. Blue and red circles in panels (**A**,**C**) represent non-tumor and tumor/HCC samples, respectively. In panel (**B**), blue and red circles represent matched adjacent non-tumor and tumor samples, respectively, and gray lines connect paired samples from the same patient. Blue circles in panel (**D**) represent individual tumor samples within each histologic-grade group. In panel (**E**), light-blue, red, green, and purple circles represent individual biological replicates for THLE-2, MHCC97-H, HepG2, and Huh-7 cells, respectively. Boxes denote the interquartile range, center lines indicate the median, and whiskers extend to values within 1.5 times the interquartile range. Data in panel (**E**) are presented as mean ± SD from three independent biological experiments, each with two technical replicates. Panels (**A**,**C**) were analyzed using two-sided Wilcoxon rank-sum tests, panel (**B**) using a two-sided Wilcoxon signed-rank test, panel (**D**) using the Kruskal–Wallis test, and panel (**E**) using one-way ANOVA followed by Dunnett’s multiple-comparisons test, with THLE-2 as the reference group. The densitometric data presented in Supplementary Figure S7 were analyzed using one-way ANOVA followed by Dunnett’s multiple-comparisons test. Exact or threshold *p* values are shown.

Analysis of the TCGA-LIHC dataset showed that *TFPT* transcript abundance was higher in 371 primary HCC tissues than in 50 adjacent non-tumor liver tissues (*p* < 0.001; Figure 1A). This difference was also observed in the paired analysis of 50 matched tumor and adjacent non-tumor tissue samples (*p* < 0.001; Figure 1B).

The expression pattern was independently evaluated in the GSE14520-GPL3921 cohort. *TFPT* expression was higher in 225 HCC tissues than in 220 non-tumor liver tissues (*p* < 0.001; Figure 1C). Within TCGA-LIHC, *TFPT* expression differed among the four histologic-grade groups (Kruskal–Wallis *p* < 0.001; Figure 1D), although no consistent monotonic increase was observed from G1 to G4.

RT-qPCR showed higher *TFPT* mRNA expression in MHCC97-H (*p* < 0.001), HepG2 (*p* = 0.030), and Huh-7 cells (*p* < 0.001) than in THLE-2 cells (Figure 1E). Representative immunoblots showed higher TFPT protein abundance in the three HCC cell lines than in THLE-2 cells. Densitometric analyses from three independent biological experiments confirmed significantly higher relative TFPT protein abundance in MHCC97-H, HepG2, and Huh-7 cells than in THLE-2 cells (all Dunnett-adjusted *p* < 0.001; Figure 1F and Figure S7).

### 3.2. Higher TFPT Expression Was Associated with Shorter Overall Survival in TCGA-LIHC, but the Association Was Not Reproduced in GSE14520

**Figure 2:**
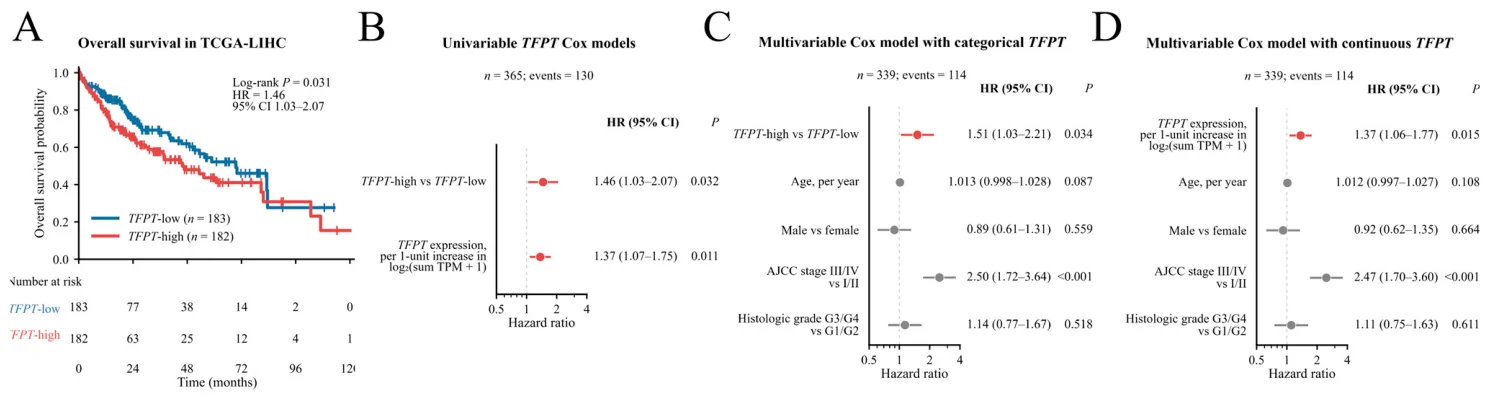
Association between tumor *TFPT* expression and overall survival in TCGA-LIHC. (**A**) Kaplan–Meier overall survival curves for the *TFPT*-low (*n* = 183) and *TFPT*-high (*n* = 182) groups, defined using the median tumor *TFPT* expression value. Curves were compared using the log-rank test. The displayed HR and 95% CI were obtained from the corresponding univariable Cox model. Numbers at risk are shown at 24-month intervals. (**B**) Univariable Cox models evaluating *TFPT* as either a median-dichotomized variable or a continuous variable per 1-unit increase in log_2_(sum TPM + 1) (365 patients; 130 deaths). (**C**) Multivariable Cox model evaluating categorical *TFPT* expression after adjustment for age, sex, AJCC stage, and histologic grade (339 complete cases; 114 deaths). (**D**) Multivariable Cox model evaluating continuous *TFPT* expression using the same covariates and sample set. Blue and red curves in panel (**A**) represent the *TFPT*-low and *TFPT*-high groups, respectively. In panel (**B**), red points and horizontal lines represent the *TFPT* terms. In panels (**C**,**D**), red points and horizontal lines represent the *TFPT* term, whereas gray points and horizontal lines represent the other covariates. Points indicate HRs, horizontal lines indicate 95% CIs, and the dashed vertical line denotes an HR of 1. Two-sided *p* values are shown. AJCC, American Joint Committee on Cancer; CI, confidence interval; HR, hazard ratio.

The TCGA-LIHC survival analysis included 365 patients with available overall survival data, among whom 130 deaths occurred. Based on the median tumor *TFPT* expression value, 183 patients were assigned to the *TFPT*-low group and 182 to the *TFPT*-high group. Overall survival was shorter in the *TFPT*-high group than in the *TFPT*-low group (log-rank *p* = 0.031; Figure 2A).

In the univariable Cox model, the hazard ratio for *TFPT*-high versus *TFPT*-low tumors was 1.46 (95% CI, 1.03–2.07; *p* = 0.032). When *TFPT* expression was analyzed as a continuous variable, the hazard ratio per 1-unit increase in log_2_(sum TPM + 1) was 1.37 (95% CI, 1.07–1.75; *p* = 0.011; Figure 2B).

Multivariable analyses included 339 complete cases and 114 death events. After adjustment for age, sex, AJCC stage, and histologic grade, the hazard ratio for *TFPT*-high versus *TFPT*-low tumors was 1.51 (95% CI, 1.03–2.21; *p* = 0.034; Figure 2C). In the corresponding continuous model, the adjusted hazard ratio per 1-unit increase in *TFPT* expression was 1.37 (95% CI, 1.06–1.77; *p* = 0.015; Figure 2D). AJCC stage was also associated with overall survival, whereas age, sex, and histologic grade were not statistically significant in the adjusted models.

The survival association was additionally evaluated in GSE14520. In the platform-adjusted multivariable model comprising 225 patients and 86 deaths, within-platform standardized *TFPT* expression was not associated with overall survival (HR per 1-SD increase, 1.02; 95% CI, 0.84–1.25; *p* = 0.830). A platform-stratified sensitivity analysis involving 242 patients and 96 deaths produced a similar nonsignificant result (HR, 1.14; 95% CI, 0.93–1.38; *p* = 0.204). Thus, the survival association observed in TCGA-LIHC was not reproduced in the available external cohort (Supplementary Figure S3).

Tests based on rank-transformed scaled Schoenfeld residuals provided no evidence of proportional-hazards violations for the *TFPT* terms or the corresponding global tests in the evaluated Cox models (all *p* ≥ 0.05; Supplementary Table S3).

### 3.3. High TFPT Expression Was Associated with Proliferative, Metabolic, and Cellular-Stress-Related Transcriptional Programs

**Figure 3:**
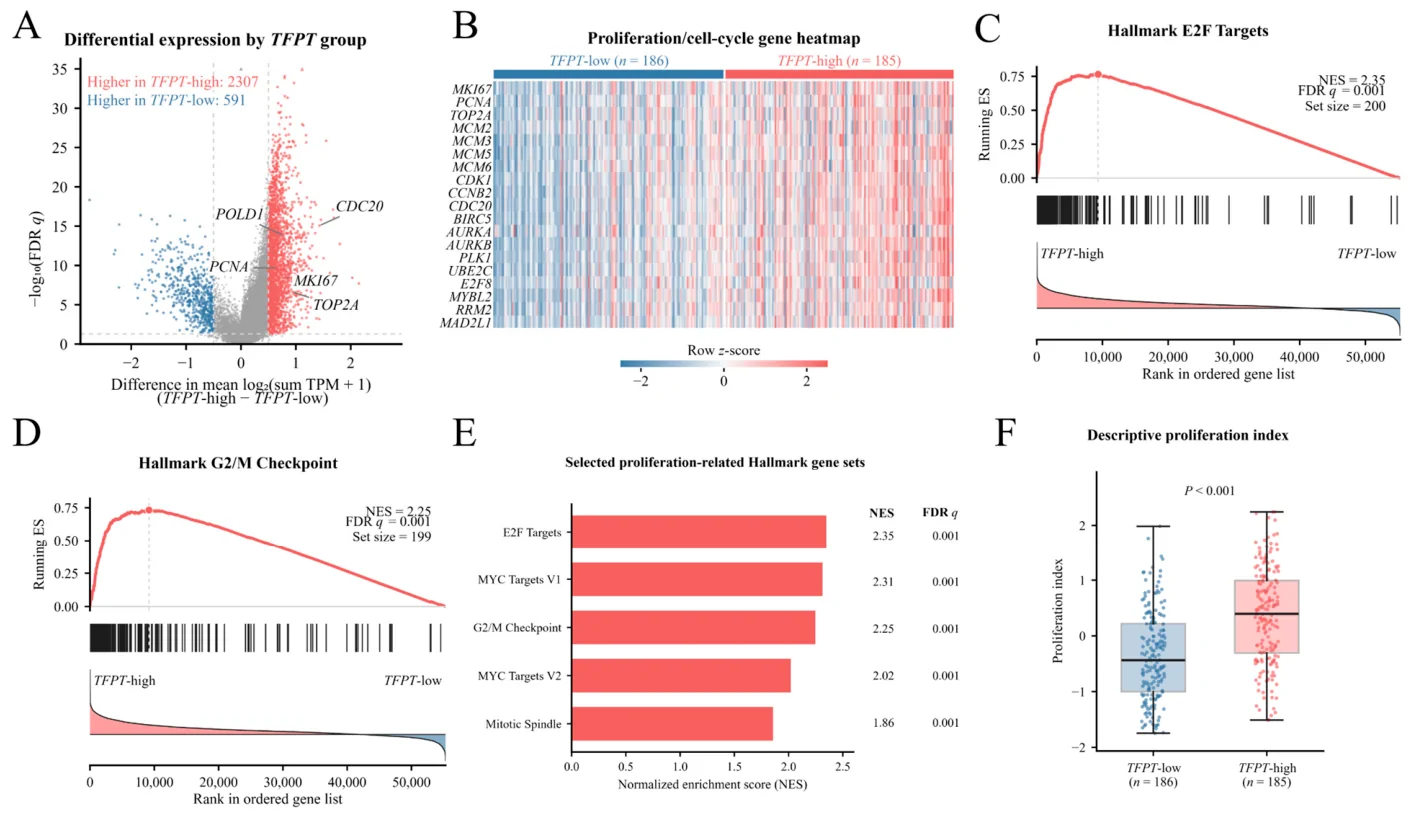
Transcriptional features associated with high *TFPT* expression in HCC. (**A**) Transcriptome-wide expression comparison of *TFPT*-high and *TFPT*-low TCGA-LIHC tumors. The *x*-axis shows the between-group difference in mean log_2_(sum TPM + 1) expression, and the *y*-axis shows −log_10_ (FDR *q*). Red and blue denote genes expressed at higher levels in *TFPT*-high and *TFPT*-low tumors, respectively, whereas gray denotes genes that did not meet both prespecified criteria of FDR *q* < 0.05 and an absolute mean difference ≥0.5. Selected proliferation- and cell-cycle-related genes are labeled. Welch’s *t* tests were followed by Benjamini–Hochberg correction. (**B**) Row-scaled expression of selected proliferation- and cell-cycle-related genes in *TFPT*-low (*n* = 186) and *TFPT*-high (*n* = 185) tumors. Columns represent individual tumors, and values are shown as row *z* scores. (**C**,**D**) Preranked GSEA plots for the Hallmark E2F Targets and G2/M Checkpoint gene sets; vertical black lines indicate gene-set members. (**E**) Summary of selected Hallmark gene sets enriched in *TFPT*-high tumors. Bars indicate normalized enrichment scores, with NES and FDR *q* values shown. (**F**) Values of the descriptive proliferation index in *TFPT*-low and *TFPT*-high tumors, compared using a two-sided Wilcoxon rank-sum test. Individual observations are overlaid on boxplots. Boxes denote the interquartile range, center lines indicate the median, and whiskers extend to values within 1.5 times the interquartile range. All significantly enriched Hallmark gene sets are shown in Supplementary Figure S4, whereas the complete Hallmark GSEA output is provided in Supplementary Table S7. ES, enrichment score; FDR, false-discovery rate; GSEA, gene set enrichment analysis; NES, normalized enrichment score.

Transcriptome-wide comparison of *TFPT*-high and *TFPT*-low TCGA-LIHC tumors identified 2307 genes with higher expression in the *TFPT*-high group and 591 genes with higher expression in the *TFPT*-low group. These genes met the prespecified criteria of FDR *q* < 0.05 and an absolute difference in mean log_2_-transformed expression of at least 0.5. Several proliferation- and cell-cycle-related genes, including *MKI67*, *PCNA*, *TOP2A*, *CDC20*, and *POLD1*, were expressed at higher levels in *TFPT*-high tumors (Figure 3A).

The heatmap showed higher expression of selected genes involved in DNA synthesis, mitotic progression, and cell-cycle regulation in the *TFPT*-high group (Figure 3B). In preranked GSEA, Hallmark E2F Targets and G2/M Checkpoint were enriched toward the *TFPT*-high end of the ranked gene list, with normalized enrichment scores of 2.35 and 2.25, respectively, and FDR *q* = 0.001 for both gene sets (Figure 3C,D).

Hallmark MYC Targets V1, MYC Targets V2, and Mitotic Spindle were also enriched toward the *TFPT*-high end of the ranked list. The normalized enrichment scores for E2F Targets, MYC Targets V1, G2/M Checkpoint, MYC Targets V2, and Mitotic Spindle were 2.35, 2.31, 2.25, 2.02, and 1.86, respectively, with FDR *q* = 0.001 for all five gene sets (Figure 3E). The descriptive proliferation index was also higher in *TFPT*-high tumors than in *TFPT*-low tumors (*p* < 0.001; Figure 3F).

Analysis of the complete Hallmark collection identified 27 significantly enriched gene sets at FDR *q* < 0.05, including 25 enriched toward the *TFPT*-high end and two enriched toward the *TFPT*-low end of the ranked list. In addition to the proliferation-related programs displayed in Figure 3, gene sets enriched toward the *TFPT*-high end included Oxidative Phosphorylation (NES = 1.81, FDR *q* = 0.001), Unfolded Protein Response (NES = 1.78, FDR *q* = 0.001), PI3K–AKT–mTOR Signaling (NES = 1.76, FDR *q* = 0.001), mTORC1 Signaling (NES = 1.68, FDR *q* = 0.001), Glycolysis (NES = 1.43, FDR *q* = 0.008), and Reactive Oxygen Species Pathway (NES = 1.35, FDR *q* = 0.021). Bile Acid Metabolism (NES = −4.25, FDR *q* = 0.001) and Peroxisome (NES = −2.12, FDR *q* = 0.001) were enriched toward the *TFPT*-low end of the ranked list (Supplementary Figure S4 and Supplementary Table S7).

These findings associate high *TFPT* expression with a broader transcriptional state involving proliferative, metabolic, and cellular-stress-related programs.

### 3.4. Higher TFPT Expression Was Associated with Transcriptional Features Linked to Lipid Peroxidation Defense

**Figure 4:**
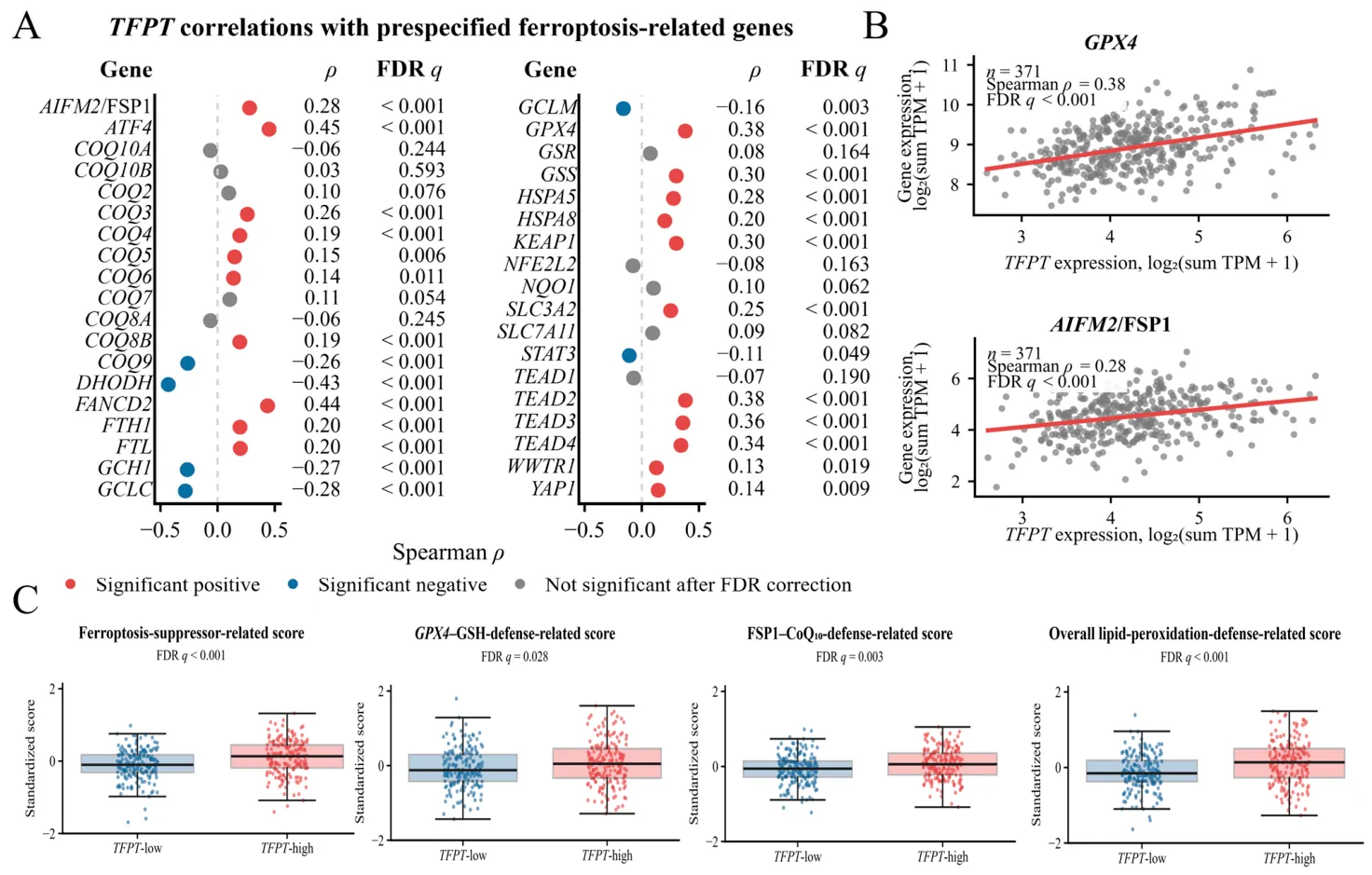
Associations between *TFPT* expression and transcriptional features linked to lipid peroxidation defense in HCC. (**A**) Spearman correlations between *TFPT* and 37 prespecified ferroptosis-related genes in 371 TCGA-LIHC tumors. Points indicate Spearman ρ values; red, blue, and gray indicate significant positive, significant negative, and nonsignificant correlations, respectively, after Benjamini–Hochberg correction. Corresponding ρ and FDR *q* values are shown. (**B**) Associations of *TFPT* with *GPX4* and *AIFM2* expression in the same cohort. *AIFM2* encodes FSP1. Each point represents one tumor. Lines show linear fits for visualization, whereas coefficients and significance values were obtained using Spearman rank-correlation analysis. Expression is shown as log_2_(sum TPM + 1). (**C**) Standardized ferroptosis-suppressor-related, GPX4–GSH-defense-related, FSP1–CoQ_10_-defense-related, and overall lipid-peroxidation-defense-related scores in *TFPT*-low (*n* = 186) and *TFPT*-high (*n* = 185) tumors. Individual observations are overlaid on separate boxplots. Two-sided Wilcoxon rank-sum tests were followed by Benjamini–Hochberg correction across the four scores. Boxes denote the interquartile range, center lines indicate the median, and whiskers extend to values within 1.5 times the interquartile range. Leave-one-gene-out analyses and external score evaluations are presented in Supplementary Figures S5 and S6, respectively. FDR, false-discovery rate; FSP1, ferroptosis suppressor protein 1; GPX4, glutathione peroxidase 4; GSH, glutathione; CoQ_10_, coenzyme Q_10_.

Given the enrichment of metabolic and cellular-stress-related programs in *TFPT*-high tumors, we next examined whether *TFPT* expression was associated with prespecified genes and descriptive expression scores linked to lipid peroxidation defense. Associations between *TFPT* expression and 37 prespecified ferroptosis-related genes were examined in 371 TCGA-LIHC tumors. Among the correlations that remained significant after multiple-testing correction, *TFPT* was positively correlated with *ATF4*, *GPX4*, *GSS*, *AIFM2*, and *SLC3A2* and negatively correlated with *GCLC*, *GCLM*, *COQ9*, *DHODH*, and *GCH1* (Figure 4A). The strongest positive correlation was observed between *TFPT* and *ATF4* (Spearman ρ = 0.45, FDR *q* < 0.001; Supplementary Figure S1).

Among selected genes related to lipid peroxidation defense, *TFPT* expression was positively correlated with *GPX4* (Spearman ρ = 0.38, FDR *q* < 0.001) and *AIFM2*, which encodes FSP1 (Spearman ρ = 0.28, FDR *q* < 0.001; Figure 4B). The association between *TFPT* and *SLC7A11* was weaker and did not remain statistically significant after correction for multiple testing (Spearman ρ = 0.09, FDR *q* = 0.082; Supplementary Figure S2).

All four internally constructed expression scores were higher in *TFPT*-high tumors than in *TFPT*-low tumors. The ferroptosis-suppressor-related score had a Cliff’s delta of 0.256 (95% CI, 0.140–0.368; FDR *q* < 0.001), whereas the GPX4–GSH-defense-related score had a Cliff’s delta of 0.132 (95% CI, 0.015–0.244; FDR *q* = 0.028). The FSP1–CoQ_10_-defense-related score had a Cliff’s delta of 0.183 (95% CI, 0.067–0.295; FDR *q* = 0.003), and the overall lipid-peroxidation-defense-related score had a Cliff’s delta of 0.266 (95% CI, 0.149–0.378; FDR *q* < 0.001; Figure 4C).

Leave-one-gene-out analyses retained a positive *TFPT*-high versus *TFPT*-low effect direction for all 57 recalculated scores, with no direction reversals. All recalculated ferroptosis-suppressor-related, FSP1–CoQ_10_-defense-related, and overall lipid-peroxidation-defense-related scores remained statistically significant after global Benjamini–Hochberg correction across all 57 leave-one-gene-out tests. For the GPX4–GSH-defense-related score, six of the nine recalculated scores remained significant after global correction, whereas removal of *GPX4*, *GSS*, or *SLC3A2* resulted in nonsignificant comparisons. These findings support directional robustness, although the statistical support for the GPX4–GSH-defense-related score was more sensitive to the contribution of individual genes (Supplementary Figure S5).

External evaluation in 247 GSE14520 tumors with available expression data showed limited and directionally inconsistent reproducibility of the TCGA-LIHC score associations. After adjustment for microarray platform, standardized *TFPT* expression was inversely associated with the FSP1–CoQ_10_-defense-related score (β = −0.165; 95% CI, −0.288 to −0.041; FDR *q* = 0.036). The association with the GPX4–GSH-defense-related score was nominally significant but did not remain significant after multiple-testing correction (β = −0.127; 95% CI, −0.251 to −0.003; *p* = 0.044; FDR *q* = 0.089).

The ferroptosis-suppressor-related score (β = −0.034; 95% CI, −0.159 to 0.091; FDR *q* = 0.765) and the overall lipid-peroxidation-defense-related score (β = 0.019; 95% CI, −0.106 to 0.144; FDR *q* = 0.765) were not associated with *TFPT* expression. No *TFPT*-by-platform interaction remained statistically significant after multiple-testing correction. Thus, the positive score associations observed in TCGA-LIHC were not uniformly reproduced in GSE14520 (Supplementary Figure S6). Additional gene-coverage-matched and leave-one-gene-out sensitivity analyses did not identify an obvious score-construction explanation for this limited external reproducibility (Supplementary Figure S6).

These composite scores summarize relative expression patterns and were not interpreted as direct measurements of ferroptosis, functional defense against lipid peroxidation, or molecular regulation by *TFPT*.

### 3.5. TFPT-Knockdown and TFPT-Overexpression Models Showed Opposing Growth-Related Phenotypes

**Figure 5:**
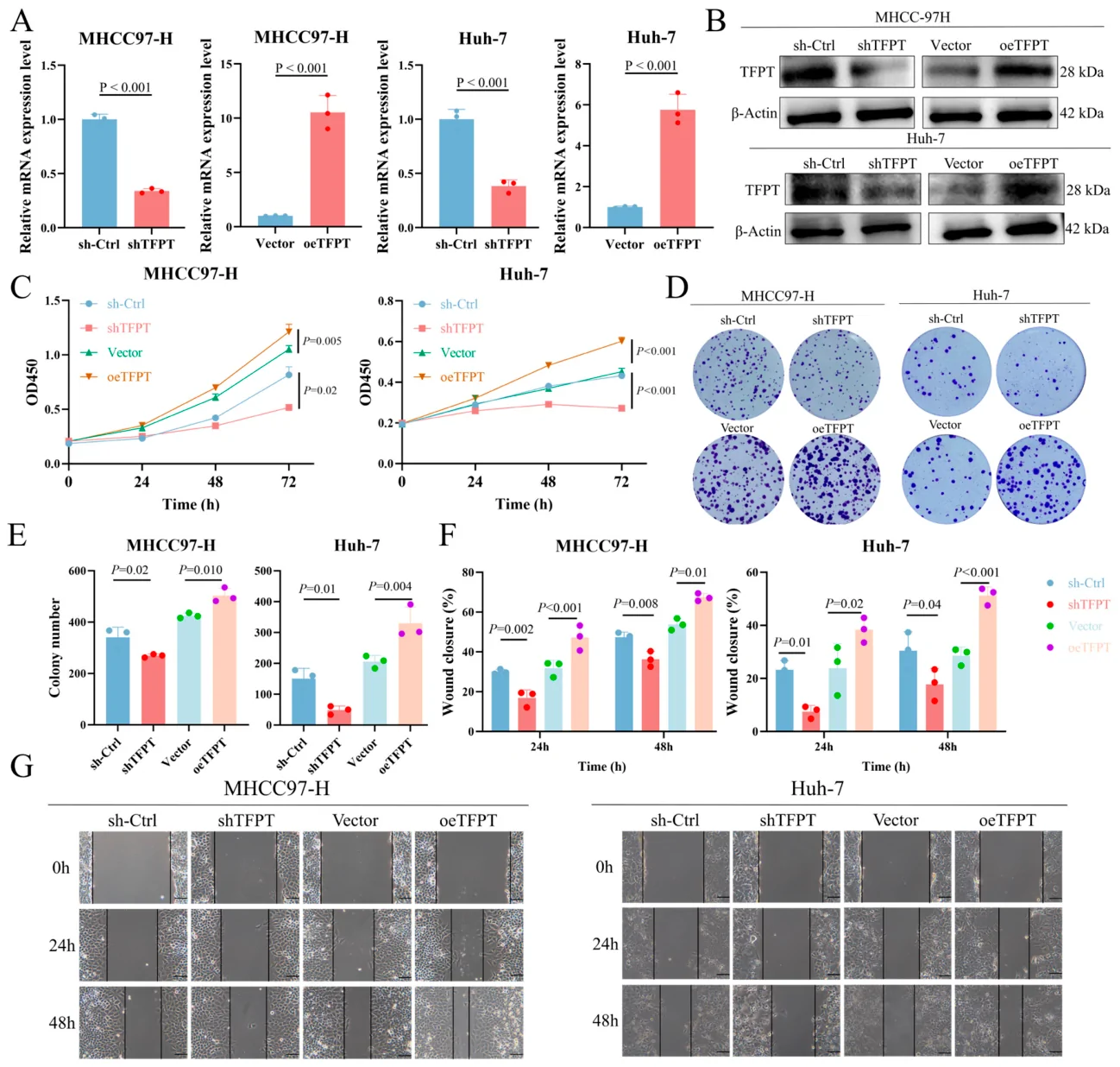
Growth-related phenotypes in *TFPT*-knockdown and *TFPT*-overexpression HCC cell models. (**A**) RT-qPCR evaluation of *TFPT* expression in stable *TFPT*-knockdown and *TFPT*-overexpression models of MHCC97-H and Huh-7 cells. (**B**) Representative TFPT immunoblots; β-actin served as the loading control. Knockdown and overexpression samples were obtained from separate membranes and were quantified and analyzed as independent comparisons. No direct densitometric comparison was performed between the two experimental models. Densitometric analyses from three independent biological experiments are presented in Supplementary Figure S8, and the corresponding full-length, uncropped images are provided in Supplementary File S2. (**C**) CCK-8 OD450 values at 0, 24, 48, and 72 h, measured using separate wells at each time point. Knockdown and overexpression datasets were analyzed separately within each cell line. Displayed *p* values correspond to the prespecified comparisons of sh-Ctrl versus shTFPT and Vector versus oeTFPT at 72 h. (**D**) Representative colony-formation images. (**E**) Colony counts. (**F**) Monolayer closure at 24 and 48 h. (**G**) Representative scratch-wound images at 0, 24, and 48 h; scale bar, 100 μm. Data in panels (**A**,**C**,**E**,**F**) are presented as individual biological-replicate values with mean ± SD from three independent biological experiments. Technical replicates, when included, were averaged within each experiment. Panels (**A**,**E**,**F**) were analyzed using two-sided unpaired Student’s *t* tests for prespecified comparisons. Panel (**C**) was analyzed using ordinary two-way ANOVA, performed separately for the knockdown and overexpression datasets within each cell line, followed by Šídák correction. Western blot densitometry was analyzed separately for each cell line and experimental model using two-sided paired Student’s *t* tests, with pairing by independent biological experiment. Exact or threshold *p* values are shown.

Stable polyclonal *TFPT*-knockdown and *TFPT*-overexpression cell populations were established in MHCC97-H and Huh-7 cells. RT-qPCR showed lower *TFPT* mRNA expression in shTFPT cells than in sh-Ctrl cells and higher expression in oeTFPT cells than in Vector controls (Figure 5A).

Representative immunoblots showed corresponding differences in TFPT protein abundance. Densitometric analyses from three independent biological experiments showed significantly lower relative TFPT protein abundance in shTFPT cells than in sh-Ctrl cells and significantly higher abundance in oeTFPT cells than in Vector controls in both cell lines. In MHCC97-H cells, the *p* values obtained using two-sided paired Student’s *t* tests were < 0.001 for sh-Ctrl versus shTFPT and 0.003 for Vector versus oeTFPT. In Huh-7 cells, the corresponding *p* values were 0.008 and 0.002, respectively (Figure 5B and Supplementary Figure S8).

At 72 h, CCK-8 signals were lower in shTFPT cells than in sh-Ctrl cells and higher in oeTFPT cells than in Vector controls in both cell lines. In MHCC97-H cells, the Šídák-adjusted *p* values were 0.020 for sh-Ctrl versus shTFPT and 0.005 for Vector versus oeTFPT. In Huh-7 cells, the adjusted *p* values for both comparisons were <0.001 (Figure 5C).

Colony numbers were lower in shTFPT cells than in sh-Ctrl cells and higher in oeTFPT cells than in Vector controls in both cell lines (Figure 5D,E). Monolayer closure at 24 and 48 h showed the same group pattern, with lower closure in shTFPT cells and higher closure in oeTFPT cells than in their corresponding controls (Figure 5F,G). Because wound closure may reflect both cell proliferation and cell displacement, these results were interpreted as a monolayer-closure-related phenotype rather than as direct evidence of altered cell migration.

### 3.6. TFPT-Knockdown and TFPT-Overexpression Models Showed Opposing Redox- and Lipid-Oxidation-Related Readouts

The relative protein abundance of GPX4, SLC7A11, and FSP1 was examined in *TFPT*-knockdown and *TFPT*-overexpression models of MHCC97-H and Huh-7 cells. Representative immunoblots showed lower band intensities for all three proteins in shTFPT cells than in sh-Ctrl cells and higher intensities in oeTFPT cells than in Vector controls.

**Figure 6:**
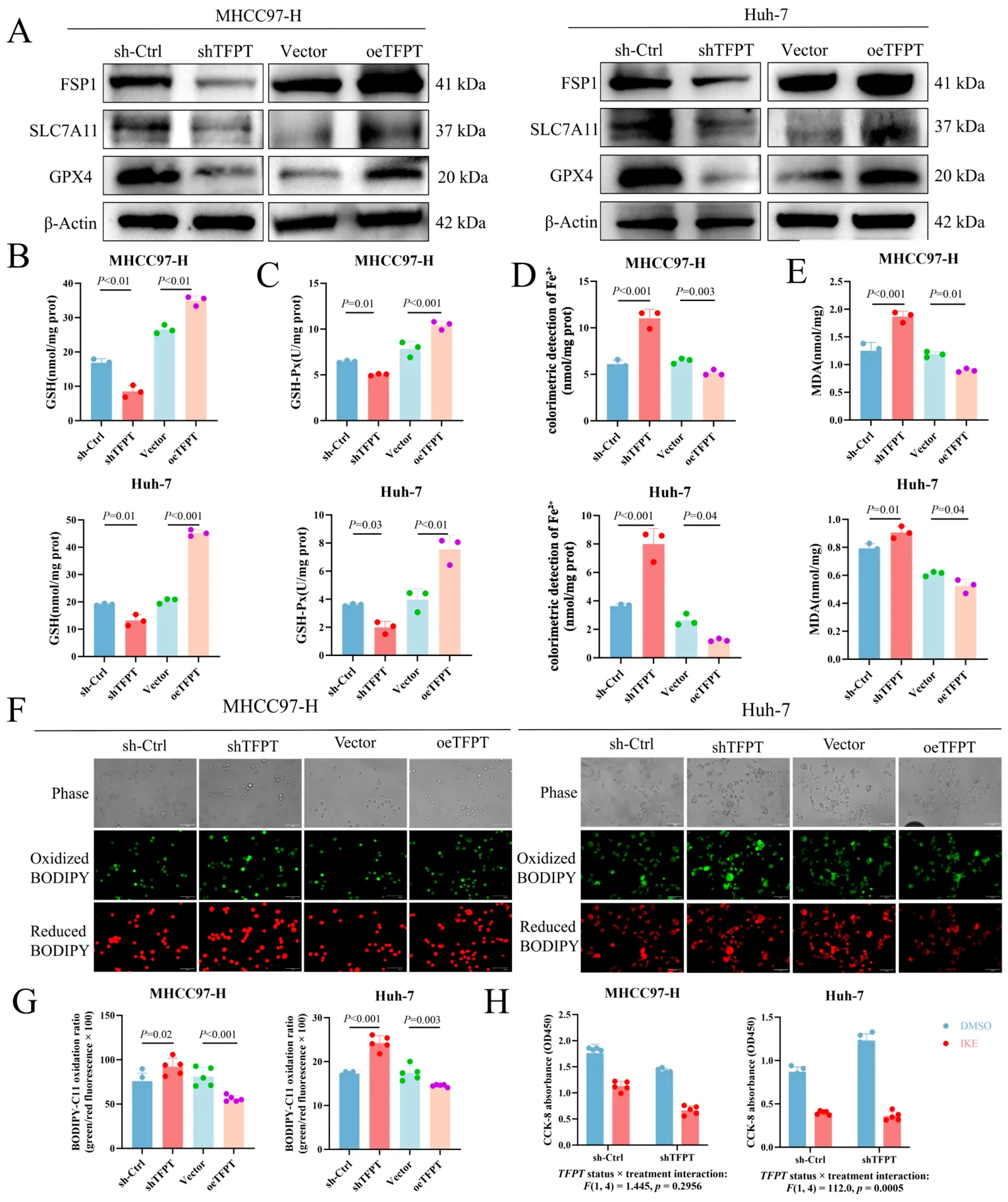
Redox- and lipid-oxidation-related readouts in *TFPT*-knockdown and *TFPT*-overexpression HCC cell models. (**A**) Representative immunoblots of GPX4, SLC7A11, and FSP1 in stable *TFPT*-knockdown and *TFPT*-overexpression models of MHCC97-H and Huh-7 cells; β-actin or GAPDH served as the loading control, as indicated. Knockdown and overexpression samples were obtained from separate membranes and were quantified and analyzed as independent comparisons. No direct densitometric comparison was performed between the two experimental models. Densitometric analyses from three independent biological experiments are presented in Supplementary Figure S9, and the corresponding full-length, uncropped images are provided in Supplementary File S2. (**B**) Intracellular GSH levels (nmol/mg protein). (**C**) Total GPx activity (U/mg protein), which was not specific to GPX4. (**D**) Colorimetrically detected intracellular Fe^2+^ levels (nmol/mg protein). (**E**) MDA levels (nmol/mg protein). (**F**) Representative phase-contrast and BODIPY-C11 581/591 images; oxidized probe is shown in green, and nonoxidized probe is shown in red. Images within each experiment were acquired using identical settings; scale bar, 100 μm. (**G**) BODIPY-C11 green-to-red fluorescence ratio, calculated as green fluorescence intensity/red fluorescence intensity × 100. In panels (**B**–**E**,**G**), blue, red, green, and purple circles represent the sh-Ctrl, shTFPT, Vector, and oeTFPT groups, respectively. (**H**) Raw CCK-8 absorbance values under DMSO or IKE treatment in *TFPT*-knockdown models. In panel (H), blue and red circles represent the DMSO- and IKE-treated conditions, respectively. Five independent biological experiments were performed, with five technical wells per condition averaged within each experiment before statistical analysis. Individual points represent biological-experiment means, and data are shown as mean ± SD. Raw biological-replicate mean OD450 values were analyzed separately for each cell line using two-way repeated-measures ANOVA, with *TFPT* status and treatment as within-experiment factors and biological experiment as the matching factor. The *TFPT*-status-by-treatment interaction was not statistically significant in MHCC97-H cells (F(1, 4) = 1.445, *p* = 0.2956) but was statistically significant in Huh-7 cells (F(1, 4) = 112.0, *p* = 0.0005). IKE/DMSO-normalized comparisons are presented separately in Supplementary Figure S10 as secondary exploratory analyses. Data in panels (**B**–**E**,**G**) are presented as individual biological-replicate values with mean ± SD from three independent biological experiments. Technical-replicate values were averaged within each experiment. Panels (**B**–**E**,**G**) were analyzed using two-sided unpaired Student’s *t* tests for the prespecified comparisons of sh-Ctrl versus shTFPT or Vector versus oeTFPT. Western blot densitometry was analyzed separately for each protein, cell line, and experimental model using two-sided paired Student’s *t* tests, with pairing by independent biological experiment.

Densitometric analyses from three independent biological experiments showed corresponding statistically significant differences in all prespecified comparisons. In the *TFPT*-knockdown models, the relative abundance of GPX4, SLC7A11, and FSP1 was lower in shTFPT cells than in sh-Ctrl cells. In the *TFPT*-overexpression models, the relative abundance of all three proteins was higher in oeTFPT cells than in Vector controls. All prespecified comparisons were statistically significant using two-sided paired Student’s *t* tests (all *p* < 0.05). Representative immunoblots are shown in Figure 6A, whereas individual biological-replicate values and exact *p* values are presented in Supplementary Figure S9.

Intracellular GSH levels and total GPx activity were lower in shTFPT cells than in sh-Ctrl cells and higher in oeTFPT cells than in Vector controls in both cell lines (Figure 6B,C). The GPx assay measured total cellular glutathione peroxidase activity and was not specific to GPX4. Intracellular Fe^2+^ and MDA levels showed the opposite group pattern, with higher values in shTFPT cells and lower values in oeTFPT cells than in their respective controls (Figure 6D,E).

Representative BODIPY-C11 images showed greater green relative to red fluorescence in shTFPT cells than in sh-Ctrl cells and lower green relative to red fluorescence in oeTFPT cells than in Vector controls. Quantification confirmed corresponding differences in the green-to-red fluorescence ratio in both MHCC97-H and Huh-7 cells (Figure 6F,G). These measurements were interpreted as lipid-oxidation-related readouts and not as direct evidence of ferroptotic cell death.

CCK-8 readouts under IKE exposure were examined only in the *TFPT*-knockdown models. The assay was performed in five independent biological experiments, with five technical-well measurements per condition averaged within each experiment. Raw biological-replicate mean OD450 values from the four experimental conditions were analyzed separately for each cell line using two-way repeated-measures ANOVA, with independent biological experiment used as the matching factor. In MHCC97-H cells, the *TFPT*-status-by-treatment interaction was not statistically significant (*F*(1, 4) = 1.445, *p* = 0.2956). In contrast, a significant interaction was observed in Huh-7 cells (*F*(1, 4) = 112.0, *p* = 0.0005), indicating that the effect of IKE on the CCK-8 readout differed according to *TFPT* status in Huh-7 cells (Figure 6H). Thus, statistical evidence of differential IKE responsiveness according to *TFPT* status was observed in Huh-7 cells but not in MHCC97-H cells.

As a secondary exploratory analysis, IKE/DMSO-normalized CCK-8 values were compared between sh-Ctrl and shTFPT cells using two-sided unpaired Student’s *t* tests. The normalized value was lower in shTFPT cells than in sh-Ctrl cells in both MHCC97-H cells (0.473 ± 0.066 versus 0.644 ± 0.076; *p* = 0.005) and Huh-7 cells (0.291 ± 0.028 versus 0.459 ± 0.021; *p* < 0.001; Supplementary Figure S10). Because the primary interaction test was statistically significant only in Huh-7 cells, the overall evidence of differential IKE responsiveness according to *TFPT* status was considered cell-line-dependent. These exploratory findings were not interpreted as evidence of ferroptotic cell death or ferroptosis-specific sensitization.

Collectively, the opposing patterns observed in *TFPT*-knockdown and *TFPT*-overexpression models associate TFPT abundance with glutathione-related antioxidant readouts, selected proteins linked to lipid peroxidation defense, intracellular Fe^2+^ and MDA levels, and lipid-oxidation-related fluorescence readouts. These findings do not establish direct molecular regulation, ferroptotic cell death, or mediation of the observed growth-related phenotypes by ferroptosis.

## 4. Discussion

The present study associates TFPT abundance with growth-related phenotypes and cellular features linked to lipid peroxidation defense in hepatocellular carcinoma (HCC). *TFPT* expression was higher in HCC tissues and selected HCC cell lines. In TCGA-LIHC, higher tumor *TFPT* expression was associated with shorter overall survival and with transcriptional programs related to proliferation, metabolism, and cellular stress. However, the survival association was not reproduced in the external GSE14520 cohort, and the internally constructed expression scores related to lipid peroxidation defense showed limited and directionally inconsistent external reproducibility. In MHCC97-H and Huh-7 cells, *TFPT*-knockdown and *TFPT*-overexpression models showed opposing patterns in CCK-8 readouts, colony formation, monolayer closure, glutathione-related measurements, intracellular Fe^2+^ and MDA levels, BODIPY-C11 green-to-red fluorescence ratios, and the relative abundance of selected proteins linked to lipid peroxidation defense. Exploratory IKE experiments further showed cell-line-dependent differential CCK-8 responsiveness according to *TFPT* status, with a significant *TFPT*-status-by-treatment interaction observed in Huh-7 but not MHCC97-H cells. Collectively, these observations associate TFPT abundance with a proliferative, stress-associated HCC state but do not establish *TFPT* as a direct regulator of ferroptosis or of any individual antioxidant pathway.

The growth-related patterns observed in the present HCC models differ from early studies of *TFPT* and its rat homolog Amida, which emphasized apoptosis and cell-cycle inhibition following ectopic expression in non-hepatic cellular contexts [14,15,16]. This difference may reflect cellular context rather than a direct contradiction. Earlier studies indicated that *TFPT*-associated apoptosis varied according to cell type, proliferative conditions, and culture density [16]. The present study used complementary stable *TFPT*-knockdown and *TFPT*-overexpression models in hepatic cancer cells rather than acute ectopic expression in non-hepatic models. Cellular origin, basal TFPT abundance, proliferative state, and the surrounding stress environment may therefore influence the phenotypes associated with *TFPT* perturbation. In MHCC97-H and Huh-7 cells, higher TFPT abundance coincided with stronger growth-related readouts rather than with a uniform pro-apoptotic or growth-suppressive phenotype.

The tumor transcriptomic analyses provided a complementary view of the cell-based findings. *TFPT*-high tumors were enriched for cell-cycle and proliferation programs, including E2F Targets, MYC Targets, G2/M Checkpoint, and Mitotic Spindle, and also showed enrichment of broader metabolic and cellular-stress-related programs. The higher descriptive proliferation index in *TFPT*-high tumors was consistent with this transcriptional pattern. Together, these findings associate higher *TFPT* expression with a broader tumor state characterized by proliferative activity, altered metabolic programs, and cellular-stress-related transcriptional features.

These transcriptomic associations remain observational. *TFPT* could contribute to the maintenance of a proliferative state, increase as a consequence of rapid proliferation, or vary together with these programs because of shared upstream regulation. GSEA cannot distinguish among these possibilities or demonstrate that *TFPT* directly activates E2F-, MYC-, mTOR-, or G2/M-related transcriptional programs. Similar restraint is required when interpreting the cell-based growth assays. CCK-8 reflects cell-associated metabolic activity and is influenced by both cell number and cellular metabolism rather than representing a direct measurement of proliferation alone. Scratch-wound closure likewise reflects both cell displacement and changes in cell number. The observed differences therefore support a monolayer-closure-related phenotype rather than a migration-specific mechanism. Direct measurements of DNA synthesis or cell-cycle progression, together with wound-closure experiments performed under proliferation-controlled conditions, would help distinguish these contributions.

Several complementary measurements supported a consistent redox-associated pattern. *TFPT*-knockdown models showed lower GSH levels and total GPx activity, together with higher Fe^2+^ and MDA levels and higher BODIPY-C11 green-to-red fluorescence ratios than their corresponding controls. *TFPT*-overexpression models showed patterns in the opposite direction. Densitometric analyses across three independent biological experiments showed corresponding differences in the relative abundance of GPX4, SLC7A11, and FSP1 in both cell lines. The convergence of biochemical, fluorescence-based, and protein-level measurements supports an association between TFPT abundance and cellular features linked to lipid peroxidation defense, but it does not identify the underlying molecular mechanism or establish a specific mode of cell death.

The SLC7A11–GSH–GPX4 and FSP1–CoQ_10_ systems act through distinct biochemical routes rather than forming a single linear pathway [5,6,7,8]. Their broadly parallel variation in the engineered cell models is therefore more consistent with a broader redox-associated cellular state than with selective regulation of one antioxidant branch. Increased proliferative and metabolic activity may be accompanied by greater oxidative and biosynthetic demands, and the observed *TFPT*-associated protein and biochemical patterns may form part of this wider cellular state. Nevertheless, the present experiments do not demonstrate that *TFPT* directly regulates the transcription, translation, stability, localization, or activity of GPX4, SLC7A11, or FSP1.

The relationship between *TFPT* and *SLC7A11* further illustrates the distinction between tumor-level transcriptional associations and cell-model protein measurements. In TCGA-LIHC, *TFPT* expression correlated positively with *ATF4*, *GPX4*, and *AIFM2*, whereas the *TFPT–SLC7A11* mRNA correlation was weak and did not remain significant after multiple-testing correction. By contrast, SLC7A11 protein abundance showed opposing differences following *TFPT* knockdown and overexpression in both engineered cell models. This protein-level result is therefore best regarded as a feature observed in both models rather than as a general transcriptional property of HCC tumors.

Several factors may contribute to the difference between tumor-level mRNA associations and cell-model protein measurements. Bulk tumor profiles contain signals from malignant, stromal, immune, and nonmalignant hepatic cells, whereas cultured cell populations provide a more homogeneous system. Post-transcriptional and post-translational processes may also separate mRNA abundance from protein abundance, localization, or activity. Previously described mechanisms involving *ATF4*, *SLC7A11*, and *GPX4* illustrate how transcript and protein behavior can diverge [9,10,11]. None of these mechanisms was directly connected to *TFPT* in the present study, and the positive *TFPT*–ATF4 correlation alone does not establish regulation. Measurements of SLC7A11 turnover, membrane localization, and cystine transport after *TFPT* perturbation would help clarify the level at which the observed protein difference arises.

The internally constructed expression scores also require cautious interpretation. In TCGA-LIHC, all four scores were higher in *TFPT*-high tumors, and leave-one-gene-out analyses retained a positive *TFPT*-high versus *TFPT*-low effect direction for all 57 recalculated scores. The ferroptosis-suppressor-related, FSP1–CoQ_10_-defense-related, and overall lipid-peroxidation-defense-related scores remained statistically significant after global Benjamini–Hochberg correction across all 57 leave-one-gene-out tests. By contrast, the GPX4–GSH-defense-related score was more sensitive to the contribution of individual genes, with six of nine recalculated scores remaining significant after global correction and removal of *GPX4*, *GSS*, or *SLC3A2* attenuating the association below the significance threshold.

More importantly, the positive score patterns observed in TCGA-LIHC were not uniformly reproduced in GSE14520. The FSP1–CoQ_10_-defense-related score showed an inverse association with *TFPT* after adjustment for microarray platform, whereas the other external associations were nonsignificant after correction or showed only nominal evidence. No *TFPT*-by-platform interaction remained significant after multiple-testing correction. Differences in gene coverage, probe representation, measurement platform, cohort composition, and biological context may have contributed to the limited external reproducibility. Notably, *AIFM2*, which encodes FSP1, was not represented in the externally reconstructed FSP1–CoQ_10_-defense-related score on either GSE14520 platform; therefore, the inverse association observed for this external score should not be interpreted as evidence of an inverse *TFPT*–FSP1 relationship. Nevertheless, the external results indicate that the positive score associations observed in TCGA-LIHC were not robustly reproduced. The scores should therefore be regarded as descriptive summaries of relative transcriptional patterns rather than as independently validated measurements of ferroptosis or functional defense against lipid peroxidation.

The IKE experiment provided a limited exploratory pharmacological assessment of CCK-8 readouts under system xc^−^ inhibition [5,12]. In the primary interaction analysis of raw biological-replicate OD450 values, the *TFPT*-status-by-treatment interaction was not statistically significant in MHCC97-H cells but was statistically significant in Huh-7 cells. Thus, statistical evidence of differential IKE responsiveness according to *TFPT* status was observed in Huh-7 cells but not in MHCC97-H cells. Although the IKE/DMSO-normalized CCK-8 values were lower in shTFPT cells than in sh-Ctrl cells in both cell lines, these ratio-based comparisons were secondary exploratory analyses, and the overall findings therefore indicate cell-line-dependent rather than uniformly reproducible IKE responsiveness. Moreover, CCK-8 readouts are influenced by both cell number and cellular metabolic activity, and the experiment evaluated only one IKE concentration, one exposure duration, and *TFPT*-knockdown models. Neither pharmacological rescue using established ferroptosis inhibitors or iron chelation nor an independent cell-death assay was included. Accordingly, these findings do not establish ferroptotic cell death or ferroptosis-specific sensitization.

The temporal relationship between the growth-related and redox-associated phenotypes remains unresolved. TFPT may primarily support or track a proliferative state, with differences in glutathione metabolism, antioxidant-protein abundance, iron handling, and lipid oxidation arising secondarily. Alternatively, TFPT may be associated with growth- and redox-related features through partly separate routes, or both phenotypes may reflect a broader cellular state. Differences in cell density, cell number, cell-cycle composition, and metabolic demand may also contribute to variation in GSH content, antioxidant-protein abundance, iron-related measurements, and lipid oxidation. Time-resolved experiments would help distinguish these possibilities. Redox-related changes preceding measurable differences in cell number would provide stronger support for a more direct relationship, whereas changes emerging only after growth divergence would be more consistent with a secondary response.

The survival analyses identified a cohort-specific association but do not support TFPT as a validated prognostic biomarker. In TCGA-LIHC, continuous *TFPT* expression remained associated with overall survival after adjustment for age, sex, AJCC stage, and histologic grade. However, the association was not reproduced in either the platform-adjusted or platform-stratified analyses of GSE14520. Differences in patient composition, disease stage, treatment, clinical annotation, transcriptomic platform, sample processing, and residual confounding may contribute to the inconsistent results. Independent cohorts with standardized RNA or protein measurements, detailed clinicopathologic information, and prespecified statistical models are required to determine whether TFPT provides prognostic information beyond established clinical variables. The current evidence does not justify TFPT-based patient stratification or treatment selection.

Several technical limitations further constrain the conclusions. The functional experiments were restricted to two HCC cell lines, used one *TFPT*-targeting shRNA, and did not include re-expression of shRNA-resistant *TFPT*. Although the opposing knockdown and overexpression patterns provide internal consistency, sequence-specific and construct-related effects cannot be excluded in the absence of independent silencing reagents and genetic rescue. Knockdown and overexpression experiments were performed as separate experimental systems and were not designed for direct quantitative comparison with each other. Stable selection may also have introduced longer-term cellular changes that cannot be distinguished from the immediate consequences of *TFPT* perturbation. Independent silencing reagents, genetic rescue, additional HCC models, and in vivo validation would strengthen the functional evidence.

Bulk RNA-sequencing and microarray data cannot distinguish tumor-cell-intrinsic expression from stromal, immune, or nonmalignant hepatic signals. The internally constructed proliferation- and lipid-peroxidation-defense-related scores were derived and initially evaluated within the same *TFPT*-focused TCGA cohort and should therefore be regarded as descriptive rather than independent functional measurements. Their limited external reproducibility further constrains their interpretation. In addition, the limited number of biological replicates and the targeted protein panel restrict the precision and generalizability of the protein-level findings.

Finally, whether ferroptotic cell death occurred remains unresolved [3,4]. MDA and the BODIPY-C11 green-to-red fluorescence ratio reflect lipid-oxidation-related changes but do not, by themselves, identify ferroptosis, and the total GPx assay was not specific to GPX4. Future studies should combine dose- and time-dependent ferroptosis-inducing conditions with pharmacological rescue, orthogonal cell-death measurements, and mechanistic analyses of SLC7A11, GPX4, FSP1, ATF4, and related regulators.

Overall, the present findings associate TFPT abundance with growth-related phenotypes and a set of cellular and transcriptional features related to redox status and linked to lipid peroxidation defense in HCC. They do not establish direct molecular regulation, ferroptotic cell death, independently validated prognostic utility, or mediation of the observed growth-related phenotypes by ferroptosis. Instead, the results provide a framework for future studies examining whether TFPT marks or contributes to a proliferative, stress-associated state in selected HCC contexts.

## 5. Conclusions

By integrating public transcriptomic and clinical datasets with stable *TFPT*-knockdown and *TFPT*-overexpression models in HCC cells, this study investigated associations between TFPT abundance and growth-related phenotypes and cellular features linked to lipid peroxidation defense. *TFPT* expression was higher in HCC tissues and selected HCC cell lines. In TCGA-LIHC, higher *TFPT* expression was associated with shorter overall survival after multivariable adjustment, and *TFPT*-high tumors showed enrichment of proliferative, metabolic, and cellular-stress-related transcriptional programs. However, the survival association was not reproduced in GSE14520, and the internally constructed expression scores related to lipid peroxidation defense showed limited and directionally inconsistent external reproducibility. In MHCC97-H and Huh-7 cells, *TFPT*-knockdown and *TFPT*-overexpression models showed opposing patterns in growth-related phenotypes, glutathione-related measurements, intracellular Fe^2+^ and MDA levels, lipid-oxidation-related readouts, and the relative abundance of selected proteins linked to lipid peroxidation defense. Collectively, these findings associate TFPT abundance with a proliferative, stress-associated state in selected HCC contexts. They do not establish direct molecular regulation, ferroptotic cell death, independently validated prognostic utility, or mediation of the observed growth-related phenotypes by ferroptosis.

## Data Availability

TCGA-LIHC RNA-sequencing data are publicly available from the NCI Genomic Data Commons, and the TCGA-LIHC Legacy clinical matrix used for the overall survival analyses is available through UCSC Xena. GSE14520 expression and clinical data generated on the GPL3921 and GPL571 platforms are publicly available from the Gene Expression Omnibus. Hallmark gene sets and ferroptosis-related database information are publicly available from MSigDB and FerrDb, respectively. Bioinformatics source-data files, definitions of the internally constructed expression scores, sample-level scores, processed data, analysis code, software environment and package-version records, data dictionaries, and quality-control reports are provided with the article. Full-length, uncropped Western blot images corresponding to Figure 1F, Figure 5B and Figure 6A are provided in Supplementary File S2. Raw target-protein and loading-control densitometric measurements underlying the quantitative analyses in Supplementary Figures S7–S9 are provided in Supplementary File S1. Numerical data underlying the remaining cell-based experiments are available from the corresponding author upon reasonable request.

## Supplementary Materials

The following supporting information can be downloaded at https://www.mdpi.com/article/10.3390/biology15161396/s1. Figure S1: Association between *TFPT* and *ATF4* expression in TCGA-LIHC; Figure S2: Association between *TFPT* and *SLC7A11* expression in TCGA-LIHC; Figure S3: Survival association in TCGA-LIHC and external evaluation in GSE14520; Figure S4: All significantly enriched Hallmark gene sets for *TFPT*-high versus *TFPT*-low TCGA-LIHC tumors; Figure S5: Leave-one-gene-out sensitivity of the four expression scores; Figure S6: Harmonized cross-cohort evaluation of the four expression scores and platform-specific sensitivity analyses in GSE14520; Figure S7: Densitometric analysis of TFPT protein abundance in THLE-2, MHCC97-H, HepG2, and Huh-7 cells; Figure S8: Densitometric analysis of TFPT protein abundance in *TFPT*-knockdown and *TFPT*-overexpression HCC cell models; Figure S9: Densitometric analysis of GPX4, SLC7A11, and FSP1 protein abundance in *TFPT*-knockdown and *TFPT*-overexpression HCC cell models; Figure S10: IKE/DMSO-normalized CCK-8 readouts in *TFPT*-knockdown HCC cell models. Table S1: RT-qPCR primers, primers used to prepare the *TFPT* overexpression construct, and shRNA oligonucleotides; Table S2: Composition and scoring definitions of the 19-gene panel used to calculate the descriptive proliferation index and the four internally constructed expression scores related to lipid peroxidation defense; Table S3: Variable-level and global proportional-hazards diagnostics; Table S4: Software environment and package versions used for the final analyses and figure preparation; Table S5: Complete univariable and multivariable Cox regression results; Table S6: Spearman correlations between *TFPT* and the 37 prespecified ferroptosis-related genes; Table S7: Complete Hallmark preranked GSEA results; Table S8: Sample manifest indicating inclusion status for each analysis. File S1: Supplementary Tables S1–S8 together with WB S7–S9 Western blot densitometry source-data worksheets underlying. Supplementary Figures S7–S9, including raw target-protein and loading-control band intensities, target/loading-control ratios, and normalized values from three independent biological experiments. File S2: Full-length, uncropped Western blot images corresponding to Figure 1F, Figure 5B and Figure 6A, including molecular-weight information and lane identities.
