# Supplementary material for: TFPT Abundance Is Associated with Growth-Related Phenotypes and Cellular Features Linked to Lipid Peroxidation Defense in Hepatocellular Carcinoma
Source: Biology (Basel). 2026 Aug 14;15(16):1396. doi: 10.3390/biology15161396 (PMC13509960; doi:10.3390/biology15161396)
Supplement: Supplementary file 1 [file biology-15-01396-s001.zip › Supplementary File S3.pdf]

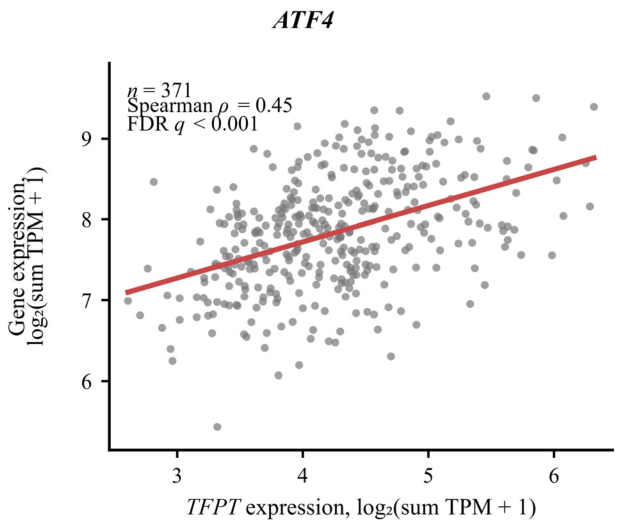

Supplementary Figure S1. Association between *TFPT* and *ATF4* expression in TCGA-LIHC.

Scatter plot of *TFPT* and *ATF4* expression in 371 TCGA-LIHC primary-tumor samples. Lines show linear fits for visualization, whereas correlation coefficients and significance values were obtained using Spearman rank-correlation analysis (rho = 0.45; Benjamini–Hochberg FDR $q < 0.001$).

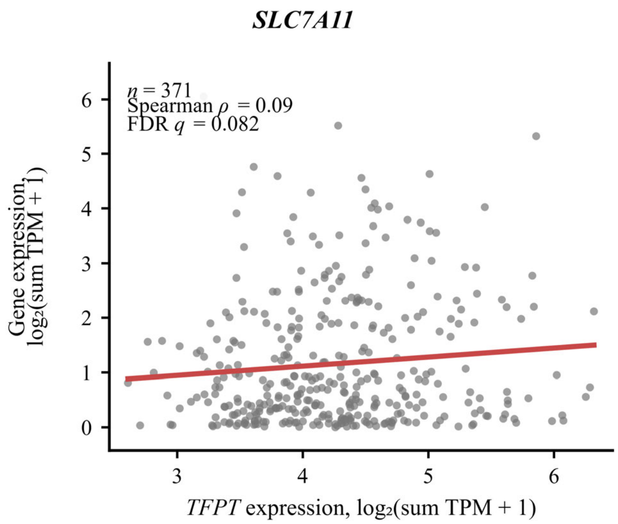

Supplementary Figure S2. Association between *TFPT* and *SLC7A11* expression in TCGA-LIHC.

Scatter plot of *TFPT* and *SLC7A11* expression in 371 TCGA-LIHC primary-tumor samples. Lines show linear fits for visualization, whereas correlation coefficients and significance values

were obtained using Spearman rank-correlation analysis (rho = 0.09; Benjamini–Hochberg FDR q = 0.082).

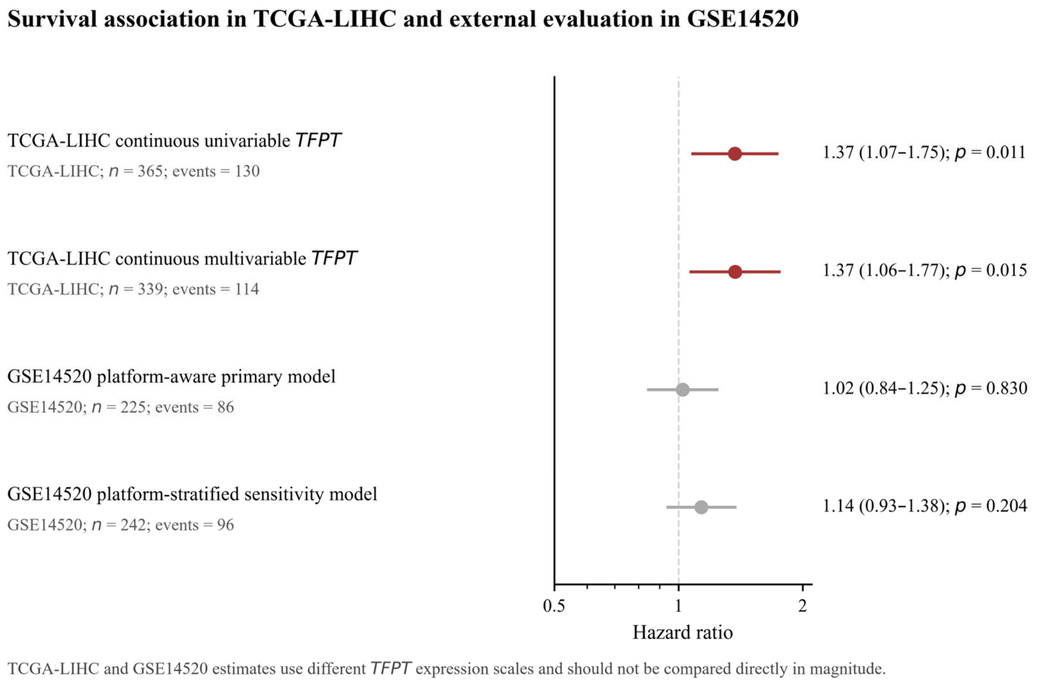

Supplementary Figure S3. Survival association in TCGA-LIHC and external evaluation in GSE14520.

Forest plot showing Cox proportional-hazards estimates. TCGA-LIHC models used 365 patients (130 events) for the continuous univariable model and 339 complete cases (114 events) for the multivariable model adjusted for age, sex, AJCC stage, and histological grade. The GSE14520 platform-aware primary model used 225 complete cases (86 events), and the platform-stratified sensitivity model used 242 cases (96 events). TCGA-LIHC estimates are reported per 1-unit increase in $\log_2$(sum TPM + 1), whereas GSE14520 estimates are reported per 1-SD increase in within-platform standardized *TFPT* expression. TCGA-LIHC and GSE14520 estimates use different *TFPT* expression scales and should not be compared directly in magnitude. Points denote hazard ratios and horizontal lines denote 95% confidence intervals. The external analyses are exploratory evaluations and did not reproduce the TCGA-LIHC survival association.

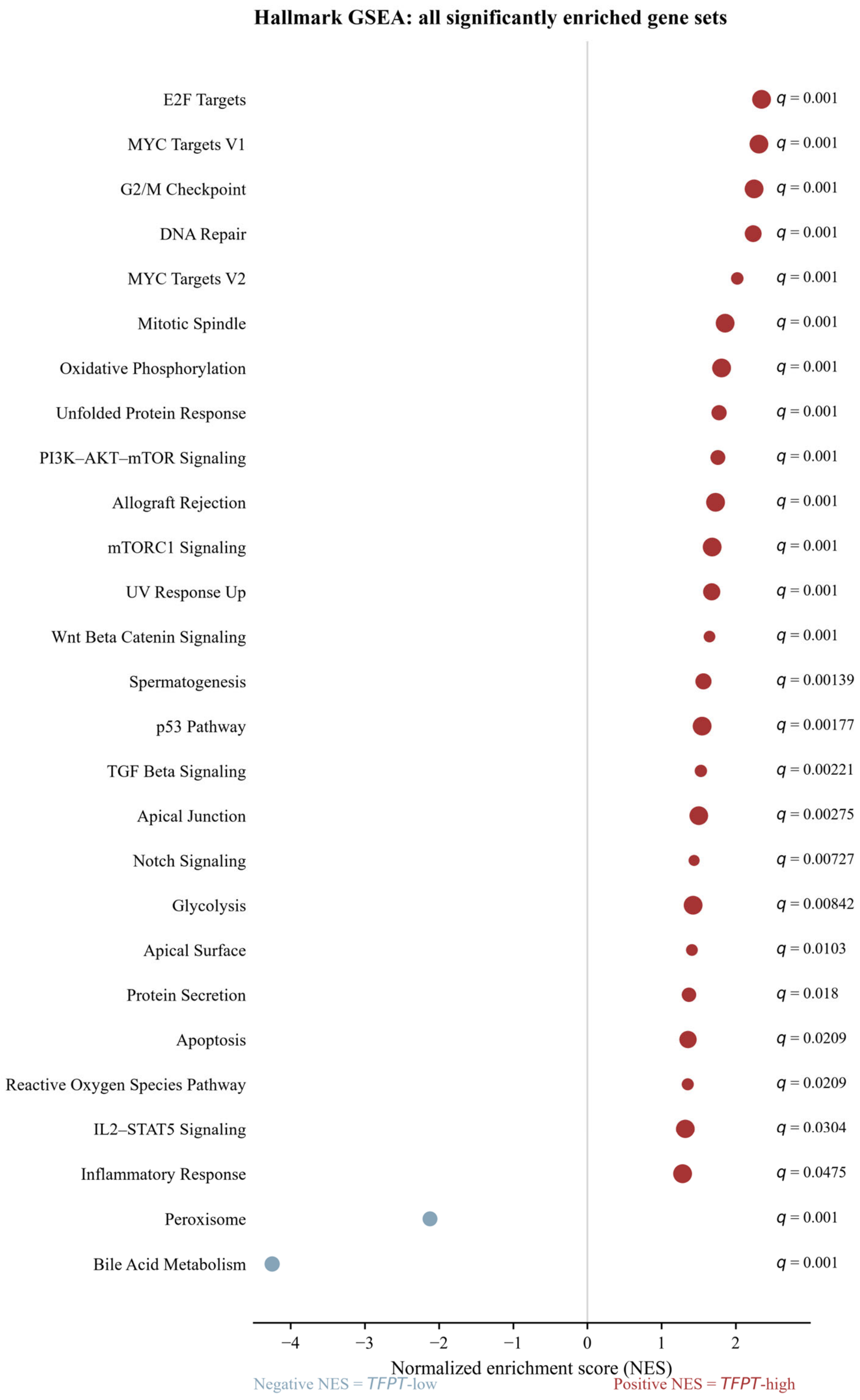

Supplementary Figure S4. All significantly enriched Hallmark gene sets for *TFPT*-high versus *TFPT*-low TCGA-LIHC tumors.

All 27 Hallmark gene sets with FDR $q < 0.05$ are shown, including 25 with positive normalized enrichment scores (NES) toward *TFPT*-high tumors and 2 with negative NES toward *TFPT*-low tumors. Positive NES values indicate enrichment toward *TFPT*-high tumors, whereas negative NES values indicate enrichment toward *TFPT*-low tumors. The complete output for all 50 Hallmark gene sets is provided in Supplementary Table S7 and the corresponding source-data file. Gene set enrichment analysis used the MSigDB Hallmark v2026.1.Hs collection, Welch t ranking, 1000 permutations, and seed 20260712.

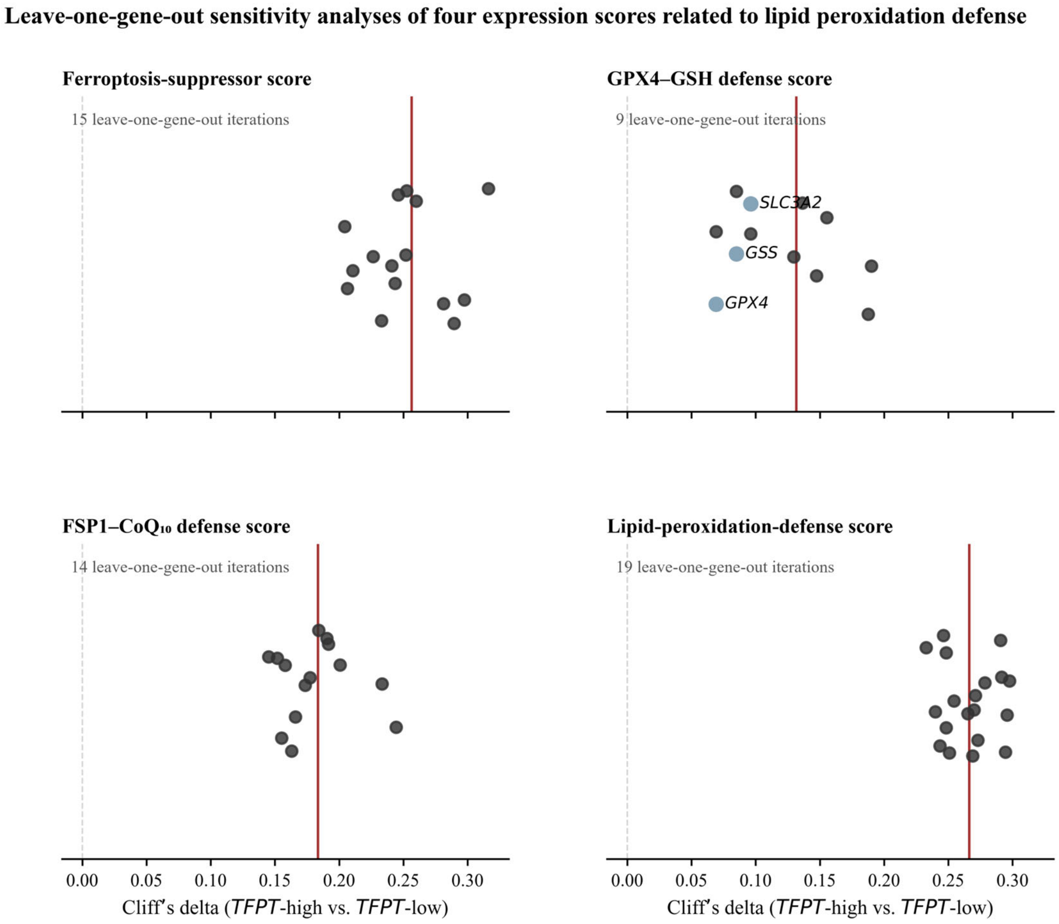

Supplementary Figure S5. Leave-one-gene-out sensitivity of the four expression scores.

Leave-one-gene-out sensitivity analyses were performed for the four internally constructed expression scores in 371 TCGA-LIHC primary-tumor samples. The red vertical line indicates the Cliff's delta obtained using the corresponding complete score. In the GPX4–GSH-defense-related score panel, blue points indicate leave-one-gene-out comparisons that did not remain statistically significant after global Benjamini–Hochberg correction across all 57 tests. These points correspond to omission of GPX4, GSS, or SLC3A2. All 57 recalculated scores retained a positive *TFPT*-high versus *TFPT*-low effect direction.

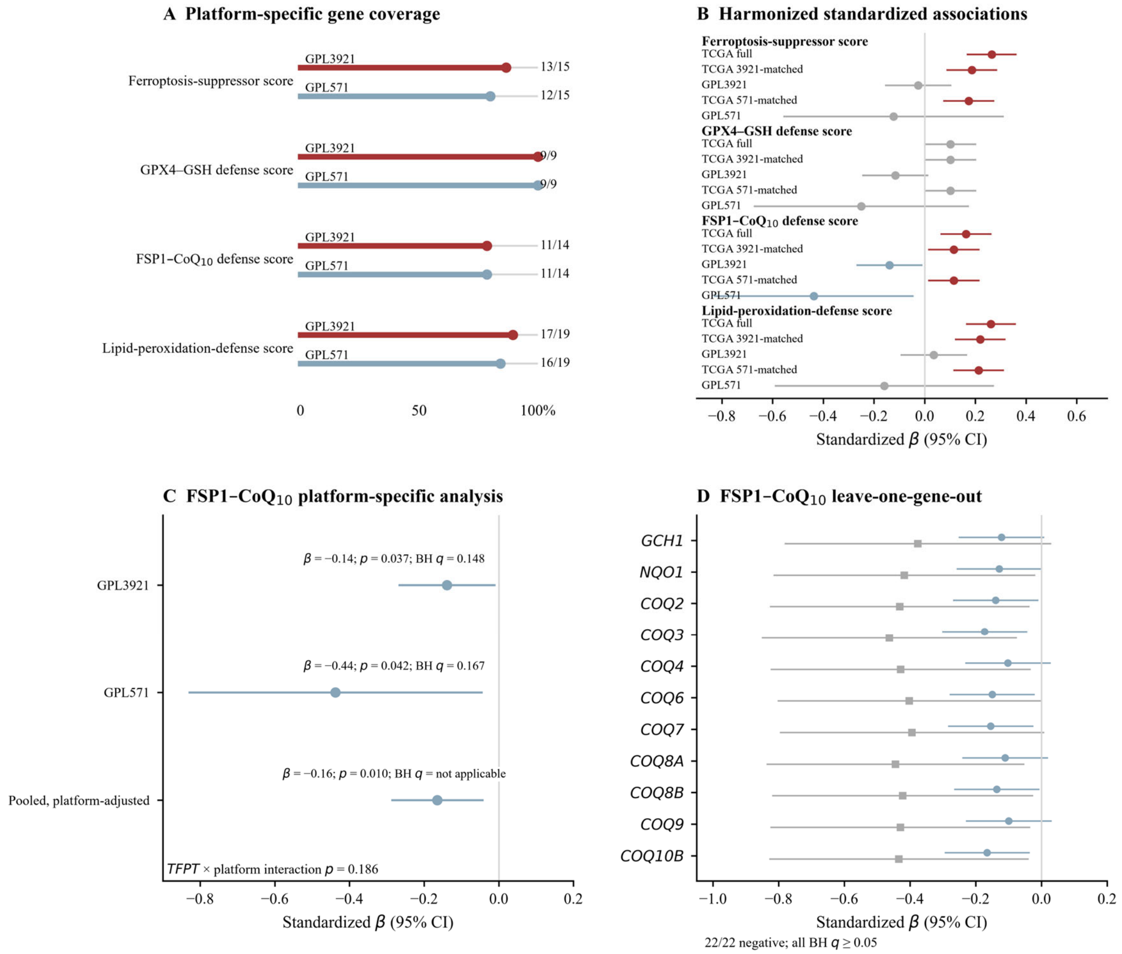

Supplementary Figure S6. Harmonized cross-cohort evaluation of the four expression scores and platform-specific sensitivity analyses in GSE14520.

(A) Platform-specific gene coverage for the four expression scores on GPL3921 and GPL571; red circles denote GPL3921 and blue circles denote GPL571. (B) Harmonized standardized associations between *TFPT* expression and the four expression scores in TCGA-LIHC and GSE14520. "TCGA GPL3921-matched" and "TCGA GPL571-matched" denote TCGA-LIHC scores recalculated using only the score genes represented on the corresponding GSE14520 microarray platform; these labels refer to gene-coverage matching rather than patient matching. Red denotes a positive estimate meeting the prespecified significance criterion, blue denotes a negative estimate meeting that criterion, and gray denotes an estimate not meeting that criterion. (C) Platform-specific and pooled platform-adjusted associations between *TFPT* expression and the FSP1–CoQ_{10}-defense-related score. Nominal P values and Benjamini–Hochberg-adjusted q values are shown for the platform-specific analyses. The *TFPT*-by-platform interaction P value is also shown. (D) External leave-one-gene-out sensitivity analyses of the FSP1–CoQ_{10}-defense-related score. Blue circles indicate GPL3921 estimates and gray squares indicate GPL571 estimates. All 22 estimates retained a negative direction, whereas none remained statistically significant after Benjamini–Hochberg correction. Points denote standardized beta estimates and horizontal lines denote 95% confidence intervals. These analyses are exploratory method-

harmonization sensitivity analyses and do not replace the prespecified TCGA-LIHC primary analysis.

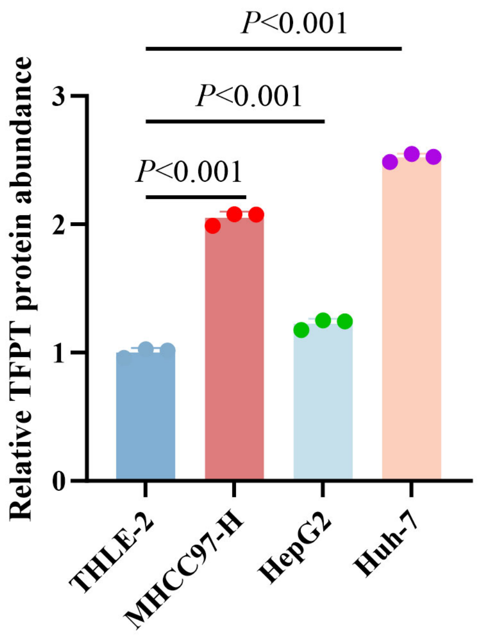

Supplementary Figure S7. Densitometric analysis of TFPT protein abundance in THLE-2, MHCC97-H, HepG2, and Huh-7 cells.

Densitometric quantification of TFPT protein abundance corresponding to the representative immunoblot shown in Figure 1F. TFPT band intensity was normalized to GAPDH. Data are presented as individual biological-replicate values with mean ± SD from three independent biological experiments. Differences among cell lines were analyzed using one-way ANOVA followed by Dunnett's multiple-comparisons test, with THLE-2 cells as the reference group. Relative TFPT protein abundance was significantly higher in MHCC97-H, HepG2, and Huh-7 cells than in THLE-2 cells (all Dunnett-adjusted $P < 0.001$). TFPT band intensity was normalized to GAPDH, and the resulting TFPT/GAPDH ratio was expressed relative to the mean value of THLE-2 cells. Exact or threshold P values are shown. The corresponding full-length, uncropped Western blot images are provided in Supplementary File S2.

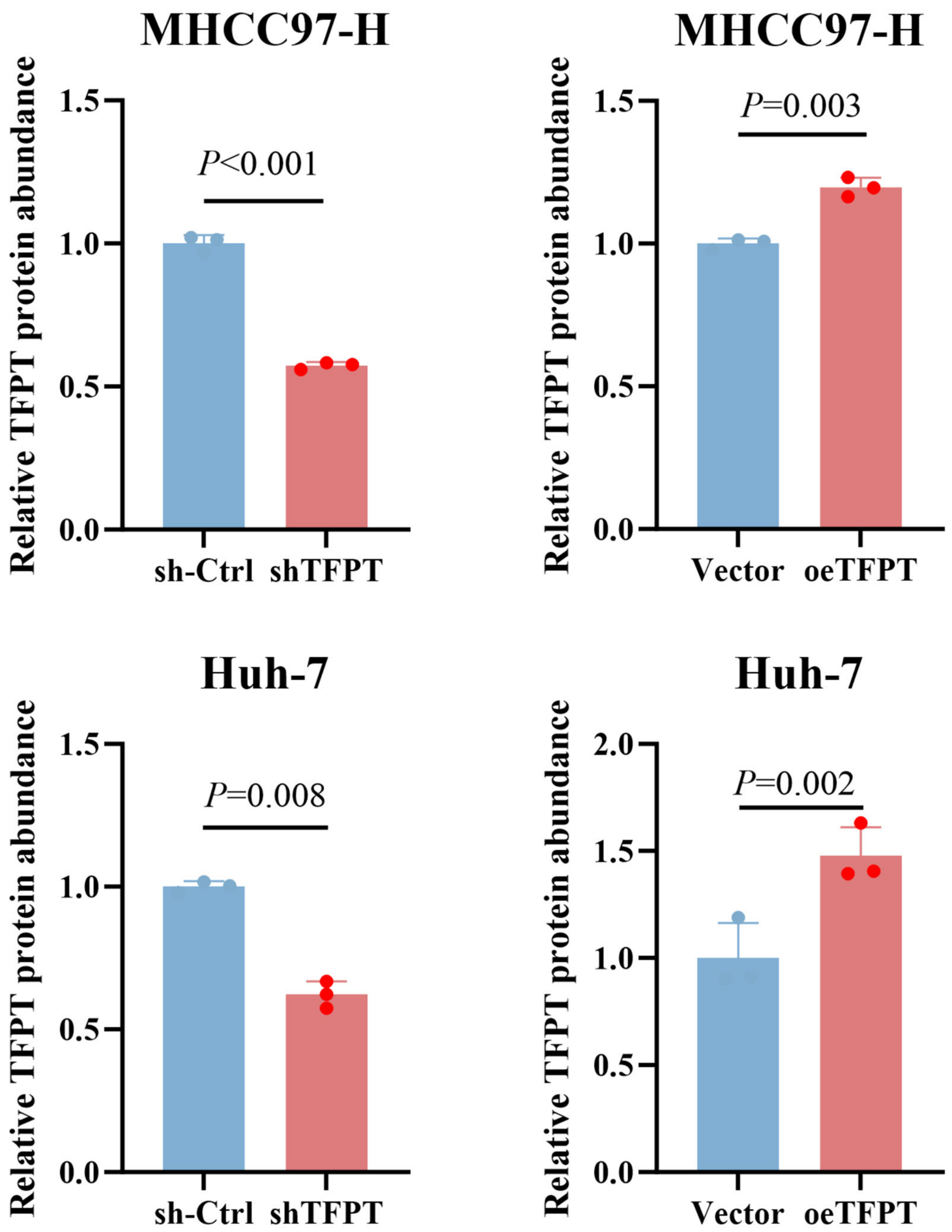

Supplementary Figure S8. Densitometric analysis of TFPT protein abundance in TFPT-knockdown and TFPT-overexpression HCC cell models.

Densitometric quantification of TFPT protein abundance corresponding to the representative immunoblots shown in Figure 5B in stable *TFPT*-knockdown and *TFPT*-overexpression models of MHCC97-H and Huh-7 cells. *β*-Actin served as the loading control. Knockdown and overexpression samples were obtained from separate membranes and were quantified and analyzed as independent comparisons; no direct densitometric comparison was performed

between the two experimental models. Data are presented as individual biological-replicate values with mean ± SD from three independent biological experiments. Analyses were performed separately for each cell line and experimental model using two-sided paired Student's t tests, with pairing by independent biological experiment. TFPT protein abundance was lower in shTFPT cells than in sh-Ctrl cells and higher in oeTFPT cells than in Vector controls in both cell lines. The paired-test P values were < 0.001 for sh-Ctrl versus shTFPT and 0.003 for Vector versus oeTFPT in MHCC97-H cells, and 0.008 and 0.002, respectively, in Huh-7 cells. The corresponding full-length, uncropped Western blot images are provided in Supplementary File S2.

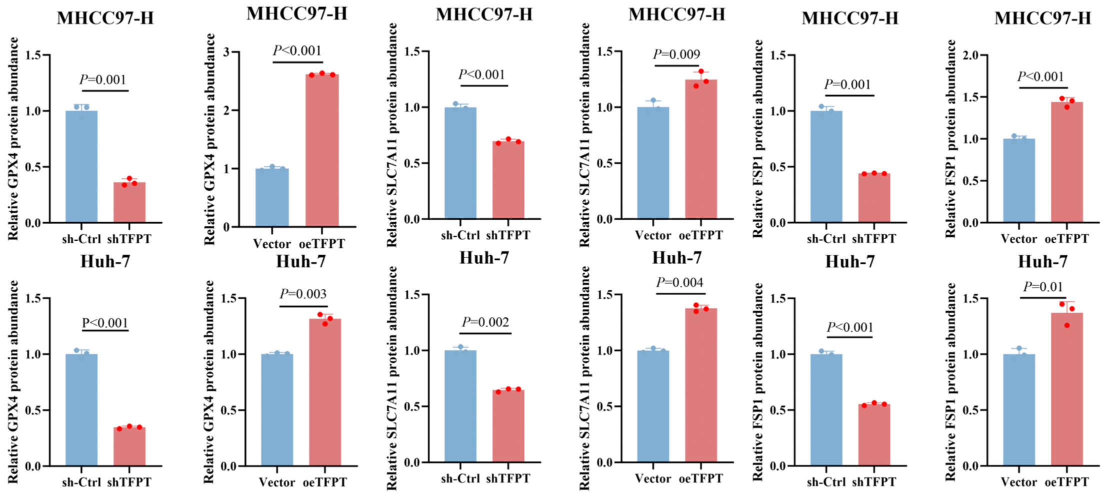

Supplementary Figure S9. Densitometric analysis of GPX4, SLC7A11, and FSP1 protein abundance in *TFPT*-knockdown and *TFPT*-overexpression HCC cell models.

Densitometric quantification of GPX4, SLC7A11, and FSP1 protein abundance corresponding to the representative immunoblots shown in Figure 6A in stable *TFPT*-knockdown and *TFPT*-overexpression models of MHCC97-H and Huh-7 cells. β-Actin or GAPDH served as the loading control, as applicable. Knockdown and overexpression samples were obtained from separate membranes and were quantified and analyzed as independent comparisons; no direct densitometric comparison was performed between the two experimental models. Data are presented as individual biological-replicate values with mean ± SD from three independent biological experiments. Densitometric analyses were performed separately for each protein, cell line, and experimental model using two-sided paired Student's t tests, with pairing by independent biological experiment. In the *TFPT*-knockdown models, the relative abundance of GPX4, SLC7A11, and FSP1 was lower in shTFPT cells than in sh-Ctrl cells, whereas in the TFPT-overexpression models, the relative abundance of all three proteins was higher in oeTFPT cells than in Vector controls. All prespecified comparisons were statistically significant (all P < 0.05). Target-protein/loading-control ratios were expressed relative to the corresponding control group (sh-Ctrl or Vector). The corresponding full-length, uncropped Western blot images are provided in Supplementary File S2.

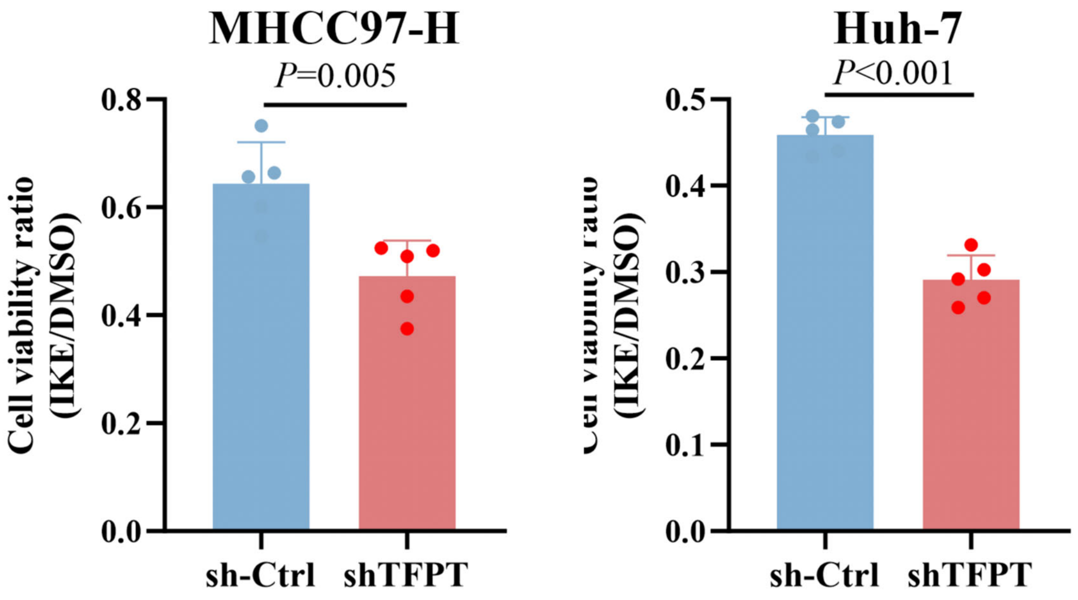

Supplementary Figure S10. IKE/DMSO-normalized CCK-8 readouts in *TFPT*-knockdown HCC cell models. CCK-8 readouts were evaluated in sh-Ctrl and shTFPT cells following treatment with 40 µM imidazole ketone erastin (IKE) or the corresponding DMSO vehicle for 24 h. Within each independent biological experiment, five technical wells per condition were averaged, and the mean OD450 value of the IKE-treated condition was normalized to that of the corresponding DMSO-treated condition. Five independent biological experiments were performed. Individual points represent independent biological experiments, and bars show mean ± SD. IKE/DMSO-normalized values were compared between sh-Ctrl and shTFPT cells using two-sided unpaired Student's t tests. The normalized CCK-8 readout was lower in shTFPT cells than in sh-Ctrl cells in both MHCC97-H ($P = 0.005$) and Huh-7 ($P < 0.001$) cells. This ratio-based analysis was considered a secondary exploratory analysis; the primary *TFPT*-status-by-treatment interaction analysis of the raw four-condition OD450 values is presented in Figure 6H.
